# Gut microbiota from green tea polyphenol-dosed mice improves intestinal epithelial homeostasis and ameliorates experimental colitis

**DOI:** 10.1186/s40168-021-01115-9

**Published:** 2021-09-07

**Authors:** Zhenhua Wu, Shimeng Huang, Tiantian Li, Na Li, Dandan Han, Bing Zhang, Zhenjiang Zech Xu, Shiyi Zhang, Jiaman Pang, Shilan Wang, Guolong Zhang, Jiangchao Zhao, Junjun Wang

**Affiliations:** 1 State Key Laboratory of Animal Nutrition, College of Animal Science and Technology, China Agricultural University, Beijing, 100193 China; 2 Key Laboratory of Animal Epidemiology of the Ministry of Agriculture, College of Veterinary Medicine, China Agricultural University, Beijing, 100193 China; 3 State Key Laboratory of Food Science and Technology, Nanchang University, Nanchang, 214122 China; 4 Department of Animal and Food Sciences, Oklahoma State University, Stillwater, OK 74078 USA; 5 Department of Animal Science, Division of Agriculture, University of Arkansas, Fayetteville, AR 72701 USA

**Keywords:** Green tea polyphenol, Colitis, Gut microbiota, Fecal microbiota transplantation, Sterile fecal filtrate

## Abstract

**Background:**

Alteration of the gut microbiota may contribute to the development of inflammatory bowel disease (IBD). Epigallocatechin-3-gallate (EGCG), a major bioactive constituent of green tea, is known to be beneficial in IBD alleviation. However, it is unclear whether the gut microbiota exerts an effect when EGCG attenuates IBD.

**Results:**

We first explored the effect of oral or rectal EGCG delivery on the DSS-induced murine colitis. Our results revealed that anti-inflammatory effect and colonic barrier integrity were enhanced by oral, but not rectal, EGCG. We observed a distinct EGCG-mediated alteration in the gut microbiome by increasing *Akkermansia* abundance and butyrate production. Next, we demonstrated that the EGCG pre-supplementation induced similar beneficial outcomes to oral EGCG administration. Prophylactic EGCG attenuated colitis and significantly enriched short-chain fatty acids (SCFAs)-producing bacteria such as *Akkermansia* and SCFAs production in DSS-induced mice. To validate these discoveries, we performed fecal microbiota transplantation (FMT) and sterile fecal filtrate (SFF) to inoculate DSS-treated mice. Microbiota from EGCG-dosed mice alleviated the colitis over microbiota from control mice and SFF shown by superiorly anti-inflammatory effect and colonic barrier integrity, and also enriched bacteria such as *Akkermansia* and SCFAs. Collectively, the attenuation of colitis by oral EGCG suggests an intimate involvement of SCFAs-producing bacteria *Akkermansia*, and SCFAs, which was further demonstrated by prophylaxis and FMT.

**Conclusions:**

This study provides the first data indicating that oral EGCG ameliorated the colonic inflammation in a gut microbiota-dependent manner. Our findings provide novel insights into EGCG-mediated remission of IBD and EGCG as a potential modulator for gut microbiota to prevent and treat IBD.

Video Abstract

**Supplementary Information:**

The online version contains supplementary material available at 10.1186/s40168-021-01115-9.

## Introduction

Inflammatory bowel diseases (IBD), clinically consisting of ulcerative colitis and Crohn’s disease, are chronic inflammatory diseases of the gastrointestinal tract [1]. They are becoming more prevalent worldwide in recent years [2]. The fact that IBD is associated with dysregulation of the gut microbiota [3, 4], and a significant loss in multiple short-chain fatty acids (SCFAs)-producing bacteria was represented in IBD patients [5]. Also, previous evidences have supported SCFAs as key molecules between the microbes and host metabolism, and SCFAs-producing bacteria attract attention [6, 7]. Recently, manipulation of the gut microbiota and its metabolites has potential for IBD therapy, such as fecal microbiota transplantation (FMT) [8, 9]. Moreover, numerous epidemiological evidences support that daily diets impact the risk in chronic diseases such as IBD [10, 11]; however, it is still unclear whether it is mediated by changes in microbiota composition.

Daily consumption of green tea, and diets rich in natural polyphenols have been linked to a reduced risk of IBD [10, 12, 13]. Green tea polyphenol Epigallocatechin-3-gallate (EGCG), a major bioactive polyphenol in green tea, possesses anti-inflammatory and anti-oxidative properties [14, 15], with known therapeutic benefits in a murine model of colitis [16, 17]. It has been reported that the gut microbiota also contributes to the metabolism of tea polyphenols to facilitate the clearance of toxic reactive metabolites in cells [18, 19]. Also, previous studies have suggested that the cross-talk between gut microbiota and host oxidative and inflammatory response is pivotal to maintain intestinal health and barrier function [20–22]. But whether the gut microbiota plays a role in EGCG-mediated alleviation of IBD is still unclear.

Considering the potential interaction of gut microbiota and EGCG, we hypothesize that attenuation of colitis by EGCG is mediated mainly through modulation of the gut microbiota, which subsequently leads to alleviation of inflammation and oxidative stress in the host [11, 23]. In this study, the alleviated effect of oral or rectal administration of EGCG was first tested in a murine model of dextran sulfate sodium (DSS)-induced colitis. Next, we modulated the gut microbial community of mice by the EGCG pre-supplementation and then explored the resistant effects in colitis. Finally, we further demonstrated the alleviated effects of specific gut microbial community from EGCG-treated donor by comparing with the mice treated with FMT and sterile fecal filtrate (SFF) from control mice.

## Materials and methods

### Animals and induction of colitis

Seven- to eight-week-old specific pathogen-free (SPF) female C57BL/6 J mice (SPF Biotechnology Co., Ltd., Beijing, China) were maintained with four animals per cage and housed in a standard SPF facility of China Agricultural University with a 12-h light and 12-h dark cycle at 22 °C. Colitis was induced by administration of DSS (molecular mass 36–40 kDa, MP Biologicals, Solon, OH, USA) through drinking water.

### Treatments and sample collection

**Fig. 1:**
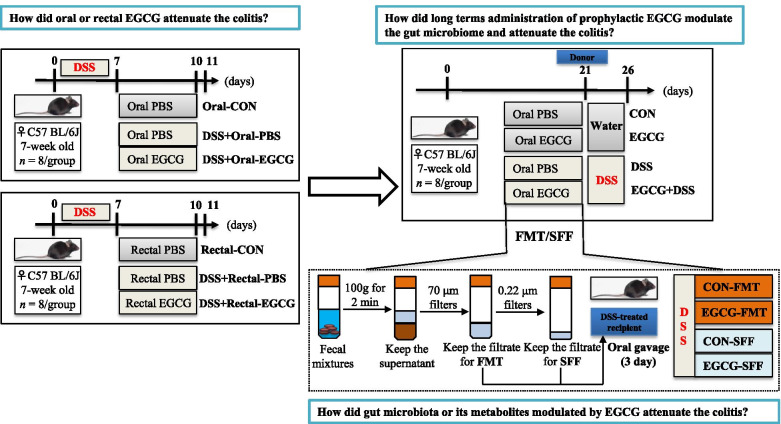
Study design for the whole experiment. EGCG, epigallocatechin-3-gallate; DSS, dextran sulfate sodium; FMT, fecal microbiota transplantation; SFF, sterile fecal filtrate

The experimental design has been shown in Fig. 1. After 1 week of acclimation, mice were randomly divided into six treatments with two cages of four mice per treatment. Animals were provided with free access to tap water supplemented with or without 2.5% DSS for 7 days, followed by daily oral or rectal administration of 100 μL PBS in the presence or absence of 50 mg/kg body weight of EGCG (purity ≥ 98%, Tokyo Chemical Industry Co., Ltd., Tokyo, Japan) for another 3 days. The treatment groups are as follows: (1) Oral-CON group: tap water for 7 days, followed by daily oral administration of PBS for 3 days; (2) DSS + Oral-PBS group: 2.5% DSS for 7 days, followed by daily oral administration of PBS for 3 days; (3) DSS + Oral-EGCG group: 2.5% DSS for 7 days, followed by daily oral administration of EGCG for 3 days; (1) Rectal-CON group: tap water for 7 days, followed by daily rectal administration of PBS for 3 days; (2) DSS + Rectal-PBS group: 2.5% DSS for 7 days, followed by daily rectal administration of PBS for 3 days; (3) DSS + Rectal-EGCG group: 2.5% DSS for 7 days, followed by daily rectal administration of EGCG for 3 days.

Body weight was measured daily for the entire duration of the study. Disease activity index (DAI) was evaluated to assess the severity of the colitis by combining scores of body weight loss, diarrhea of the stool, and the extent of blood in the feces [24, 25]. The mice were sacrificed after anesthesia on day 11, the length of the colon was measured, and fecal samples of all mice were collected sterilely and stored at − 80 °C for future analysis. Plasma was obtained by centrifugation (3000 × rpm for 15 min at 4 °C) and then stored at − 80 °C. A 5 mm segment of the mid-colon was flushed with PBS and then fixed in 10% formalin for subsequent histology analysis, while the remaining colon tissue was washed with PBS and snap frozen in liquid nitrogen for future analysis.

To study the prophylactic efficacy of EGCG in alleviating colitis, another experiment was conducted using 7- to 8-week-old SPF female C57BL/6 J mice. After 1 week of acclimation, mice were randomly divided into four groups with eight mice per group and received daily oral gavage of 100 μL PBS alone or the same volume of PBS containing 50 mg/kg body weight of EGCG for 4 weeks, with or without 2.5% DSS in drinking water for 7 days to induce acute colitis. The treatment groups are as follows: (1) CON group: daily gavage of PBS with regular tap water for 3 weeks; (2) EGCG group: daily gavage of EGCG with regular tap water for 3 weeks; (3) DSS group: daily gavage of PBS for 3 weeks with 2.5% DSS in drinking water for the last 6 days; (4) EGCG + DSS group: daily gavage of EGCG for 3 weeks with 2.5% DSS in drinking water for the last 6 days. From day 21 to day 26, body weight and DAI were measured daily. All mice were sacrificed upon anesthesia on day 27. The length of the colon was measured, and a 5-mm segment of the mid-colon was fixed in formalin for sectioning and staining. The feces and the remaining colon were sampled from each mouse and snap frozen in liquid nitrogen for future analysis. Plasma was also prepared from the blood and frozen at − 80 °C for future measurement of the cytokine concentrations. Of note, in week three, fresh feces (gut microbiota with or without its metabolites) of CON group and EGCG group were collected sterilely for subsequent FMT or SFF.

For the fecal transplants, fresh feces from each group were pooled and homogenized, diluted in sterile saline with a final concentration of 50 mg feces/mL. Pooled samples were centrifuged at 100 × g for 2 min and the supernatant was filtered through a 70 μm filters and then was used for FMT treatment. For sterile fecal filtrate (SFF), the supernatants were collected and passed through 70 and 0.22 μm filters. After 7-day drinking water with 2.5% DSS, a total of 100 μL of FMT or SFF were administered per mice via oral gavage every day.

### Histological analysis

For morphological measurements, formalin-fixed colon tissues were sectioned and stained with hematoxylin and eosin. The images were evaluated using the Image J software (US National Institutes of Health, Bethesda, MD). The extent of inflammatory infiltration, histopathological changes in crypt structure, ulceration and crypt loss, ulcer, and presence or absence of edema were measured, and the histological score was determined as previously described [24, 25]. To count colonic goblet cells, fixed colonic tissues were also stained in Alcian blue for 10–15 min and dehydrated with 100% alcohol and xylene, followed by image acquisition on a microscope (Carl Zeiss AG, Jena, Germany). Acidic mucus-containing goblet cells were counted and compared among groups.

### Terminal deoxynucleotidyl transferase-mediated dUTP nick end labeling (TUNEL) assay

The formalin-fixed colonic sections were also subjected to TUNEL staining using a TUNEL assay kit (Roche Molecular Systems, MA, Switzerland). Nuclei were stained and images were acquired using a fluorescent microscope (Carl Zeiss AG, Jena, Germany). Hoechst 33,342 and images were acquired using a fluorescent microscope (Carl Zeiss AG, Jena, Germany). The number of apoptotic cells was counted as we described earlier [26].

### Transmission electron microscopy (TEM) analysis

A 5-mm segment of fresh colon tissues was flushed with PBS and fixed in 2.5% glutaraldehyde at 4 °C for 4 h. After being rinsed in PBS, the tissue was further fixed in PBS containing 1% osmium tetroxide for 2 h at room temperature, rinsed in PBS, and dehydrated. The tissues were then embedded in Epon 812 overnight. Embedding was then performed in Epon 812 and curing was done in an oven at 60 °C for 48 h. Sections of 80 nm thickness were cut on an ultramicrotome (RMC MTX) using a diamond knife. The sections were deposited on single-hole grids coated with Formvar and carbon and double-stained in aqueous solutions of 8% uranyl acetate for 25 min at 60 °C and lead citrate for 3 min at room temperature. Images were acquired by a transmission HT7700 electron microscope (Hitachi, Tokyo, Japan).

### Quantification of inflammatory cytokines and antioxidant indexes in the colon and plasma

A portion of frozen colon samples was homogenized with RIPA lysis buffer (Solarbio, Beijing, China) to extract total proteins. The homogenate was centrifuged at 12,000 × g at 4 °C for 15 min. The protein contents were quantified using a bicinchoninic acid (BCA) protein assay kit (Solarbio, Beijing, China) according to the manufacturer’s instructions. The concentrations of pro-inflammatory cytokines such as IL-6 and TNF-α in the colon tissue were measured by ELISA (R&D Systems, Minneapolis, MN, USA) according to the manufacturers’ recommendations, while representative redox enzymes such as MPO, T-SOD, CAT, T-AOC, GSH-px, and MDA were quantified using commercial kits (Nanjing Jiancheng Bioengineering Institute, Nanjing, China). The plasma concentrations of IL-1β, IL-6, IL-8, TNF-α, T-AOC, CAT, and MDA in the plasma were also measured similarly.

### DNA extraction, 16S rRNA gene sequencing

Total microbial genomic DNA of each fecal sample was extracted using QIAamp DNA Isolation Kit (Qiagen, Hilden, Germany). The concentration and integrity of DNA were assessed using Nanodrop (Thermo Fisher Scientific, USA) and 1.5% agarose gel electrophoresis, respectively. Diluted DNA (1.0 ng/mL) was then used to amplify the V4 hypervariable region of the 16S rRNA gene with barcoded primers (515F, 5′-GTGCCAGCMGCCGCGGTAA-3′, 806R, 5′-GGACTACHVGGGTWTCTAAT-3′) and Phusion® High-Fidelity PCR Master Mix with GC Buffer (New England Biolabs, USA). PCR products were subjected to 2.0% agarose gel electrophoresis, recovered, and purified using GeneJET Gel Extraction Kit (Thermo Fisher Scientific, USA) and then pooled into equal concentrations. An equal amount of DNA was used to prepare sequencing libraries with the Ion Plus Fragment Library Kit (Thermo Fisher Scientific, USA) according to the manufacturer’s recommendations. Library quality was assessed on Qubit 2.0 Fluorometer (Thermo Fisher Scientific, USA), and sequenced on the Ion S5™ XL platform (Thermo Fisher Scientific, USA). Negative controls for DNA extraction and PCR amplification and mock community (Ion Plus Fragment Library Kit 48 rxns (Thermo Scientific, MA, USA)) were included in each HiSeq run for quality control.

### Microbiota data analysis

Raw data was analyzed using the QIIME2 platform (version 2020.2) [27]. Multiplexed single end sequencing reads (> 50,000 per sample) were imported into the QIIME2 platform. Raw reads were quality filtered, assembled, and chimeric sequences were removed using data2, which generated unique amplicon sequence variants (ASVs) instead of clustering similar sequences into traditional operational taxonomic units [28]. Subsequently, we used the SILVA reference database classifier (version 138) for the classification of ASVs with a threshold of 100% sequence similarity. Determinations of alpha and beta diversities were also conducted in QIIME 2. Principal co-ordinates analysis (PCoA) plots were generated using the “ggplot2” packages of the R software (version 3.3.1) (https://www.r-project.org/). Also, we performed permutational multivariate analysis of variance (PERMANOVA, with 999 Monte Carlo permutations) based on Bray–Curtis distances using the Adonis function in the package “vegan” in R software (version 3.3.1) [29]. Bar plots and correlations between differentially presented bacterial taxa and the concentrations of antioxidant enzymes, inflammatory cytokines, or SCFAs were calculated by Spearman correlation analysis using the “ggplot2” and “pheatmap” packages of R software (version 3.3.1), respectively (Additional file 4). Differentially abundant genera between groups were identified using linear discriminant analysis (LDA) effect size (LEfSe) analysis (http://huttenhower.sph.harvard.edu/galaxy/root?tool_id=lefse_upload) [30]. Only bacterial taxa reaching the LDA threshold of 2.0 and with average relative abundances greater than 0.01% are shown.

### Quantification of SCFAs profiles

SCFAs including acetate, propionate, and butyrate in fecal samples were quantified with ion chromatograph. Briefly, 50 mg of fecal samples were weighed, dissolved, homogenized, and then centrifuged at 3000 × g for 10 min. The supernatant was diluted (1:50), filtered through a 0.22-mm sterile membrane, kept in a 2 mL screw-cap vial, and then subjected for SCFAs analysis with an Ion Chromatography System (ThermoFisher Scientific, Waltham, Mass, USA).

### Statistical analysis

All data were represented as means ± SEM and analyzed using GraphPad Prism 8.0 program (GraphPad Software, San Diego, Canada). Data from more than two groups were compared using one-way ANOVA followed by Tukey’s multiple comparison tests. The adjusted *P* below 0.05 was considered statistically significant.

## Results

### Oral, but not rectal EGCG administration alleviated DSS-induced colitis

**Fig. 2:**
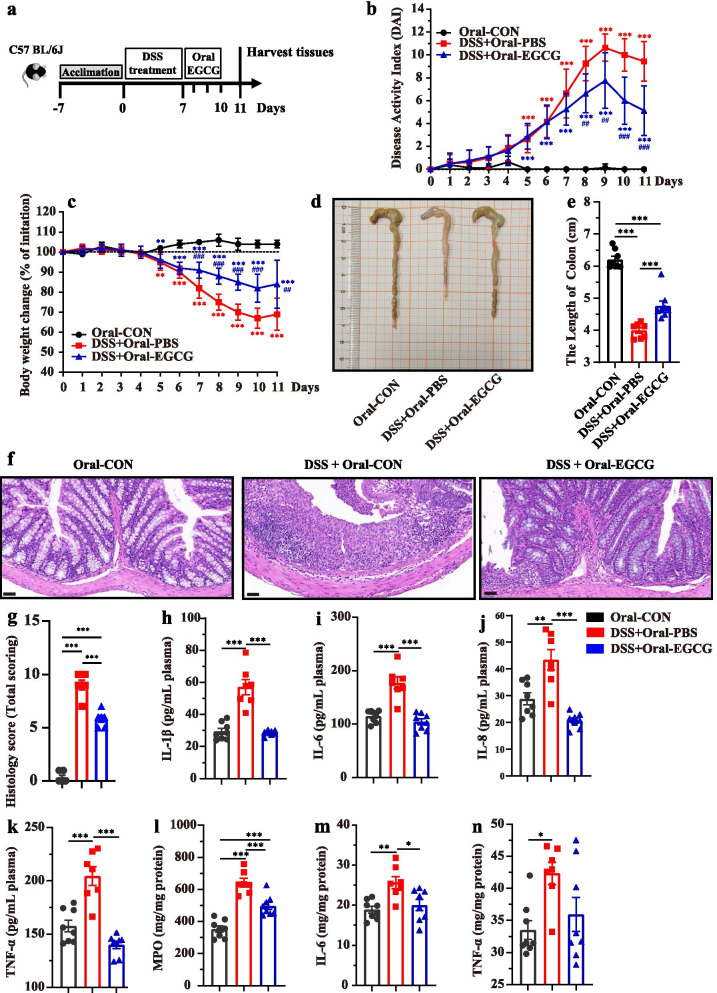
Oral EGCG alleviated DSS-induced experimental colitis. **a** Diagram illustrating the mouse model of colitis employed in this study. Oral phosphate buffer saline (PBS) and epigallocatechin-3-gallate (EGCG) treatments are indicated. **b** Kinetics of daily disease activity index (DAI) scores throughout the entire duration of the study. **c** Daily body weight changes throughout the entire duration of the study. Data were presented as means ± SEM (*n* = 7–8 per group). Statistical significance was determined using one-way ANOVA, followed by Tukey test. **P ≤ 0.01, ***P ≤ 0.001 relative to Oral-CON group; ##*P* ≤ 0.01, ###*P* ≤ 0.001 relative to DSS + Oral-PBS group. **d** Macroscopic pictures of colons and **e** the lengths of colon from each group (*n* = 7–8 per group). **f** H&E stained colon sections and **g** histological scores of colons (*n* = 6 per group). Concentrations of four representative pro-inflammatory cytokines, IL-1β (**h**), IL-6 (**i**), IL-8 (**j**), and TNF-α (**k**) in the plasma. Concentrations of myeloperoxidase (MPO) (**l**), IL-6 (**m**), and TNF-α (**n**) in the colon. Data were presented as means ± SEM (*n* = 7–8 per group). Statistical significance was determined using one-way ANOVA, followed by Tukey test. *P ≤ 0.05, **P ≤ 0.01, ***P ≤ 0.001

To study the alleviated effect of EGCG on DSS-induced colitis, experimental colitis was induced in mice by administering 2.5% DSS in water continuously for 7 days, followed by 3 days of daily oral (Fig. 2a) or rectal delivery (Figure S1a) of 50 mg/kg body weight EGCG, based on the optimal dose that we have observed previously (data not shown). To explore how oral EGCG or enteric delivery EGCG alleviated DSS-induced colitis, the symptoms of mice treated by two different ways were measured. As shown in Fig. 2b-e, compared with the DSS group, oral EGCG intervention significantly alleviated DSS-induced colitis, as evidenced by the markedly reduced disease activity index (DAI, the combined score of body weight loss, stool consistency, and rectal bleeding) score, decreased body weight loss, and relieved colon shortening. However, rectal delivery of EGCG failed to improve any of the measurements above (Additional file 1: Figure S1b, Figure S1c, Figure S1d, and Figure S1e). Histological analysis further showed obvious attenuation of inflammatory cell infiltration and mucosal damage and overall histology score in the colon in response to oral (Fig. 2f and g), but not rectal EGCG (Additional file 1: Figure S1f and Figure S1g).

To further assess the impact of EGCG on systemic and intestinal inflammatory response, myeloperoxidase (MPO) and pro-inflammatory cytokines in the plasma and colonic tissues were measured. Plasma concentrations of IL-1β (Fig. 2h), IL-6 (Fig. 2i), IL-8 (Fig. 2j), and TNF-α (Fig. 2k) were significantly decreased in DSS-treated mice in response to oral EGCG. In contrast, rectal EGCG failed to suppress these cytokines in the plasma (Additional file 1: Figure S1h, Figure S1i, Figure S1j, and Figure S1k). Similarly, DSS drastically elevated the concentration of MPO in the colon of mice, but such an elevation was significantly dampened by oral EGCG (Fig. 2l). Furthermore, both oral and rectal EGCG significantly decreased the colonic levels of IL-6 (Figs. 2m and S1m), and TNF-α showing a strong tendency to decrease as well (Fig. 2n and Additional file 1: Figure S1n). However, rectal EGCG failed to decrease the levels of MPO (Additional file 1: Figure S1l) in the colon of DSS-treated mice.

Collectively, these results indicated that clinical colitis symptoms and colonic damage were ameliorated by oral, but not rectal, EGCG.

### Oral EGCG suppressed DSS-induced oxidative stress in intestinal mucosa and improved the barrier function

**Fig. 3:**
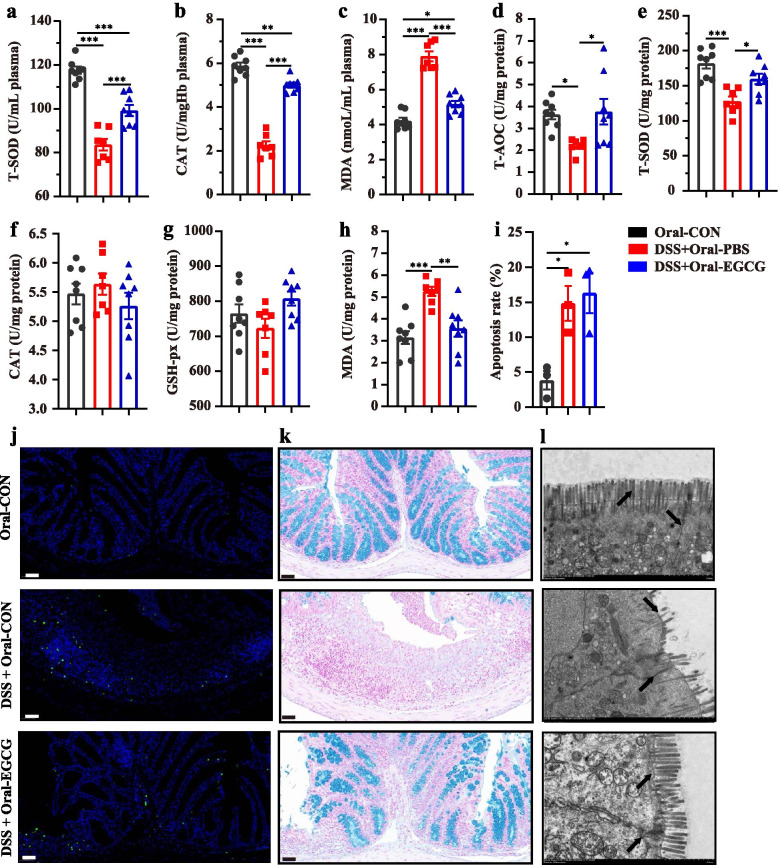
Oral EGCG attenuated oxidative stress and colonic damage. Concentrations of T-SOD (**a**), CAT (**b**), and MDA (**c**) in plasma from each group. **d** Level of T-AOC in the colon. Concentrations of T-SOD (**e**), CAT (**f**), GSH-px (**g**), and MDA (**h**) in the colon (*n* = 7–8 per group). **i** Apoptosis rate in colonic sections (*n* = 6 per group). **j** Representative fluorescent pictures of TUNEL staining of colonic sections. Scale bars represent 50 μm. **k** Representative images of Alcian blue-stained inner mucus layer of colonic sections. Scale bars represent 50 μm. **l** Representative images showing the microstructure of colonic epithelia by TEM. Data are presented as means ± SEM. Statistical significance was determined using one-way ANOVA, followed by Tukey test. *P ≤ 0.05, **P ≤ 0.01, ***P ≤ 0.001

To assess the influence of EGCG on oxidative stress, total antioxidant capacity (T-AOC), total superoxide dismutases (T-SOD), catalase (CAT), glutathione peroxidase (GSH-px), and malondialdehyde (MDA) in the plasma and colon were measured. DSS-suppressed concentrations of T-SOD and CAT, and DSS-increased level of MDA in the plasma in the mice with colitis compared with normal controls were largely attenuated by oral EGCG (Fig. 3a, b, and c). However, DSS-induced suppression of the plasma concentrations of T-SOD and CAT in the mice with colitis compared with normal controls were not obviously impacted by rectal EGCG, though the level of MDA was also decreased (Additional file 2: Figure S2a, Figure S2b, and Figure S2c). The colonic levels of T-AOC and T-SOD were significantly decreased by DSS, but attenuated to normal by oral EGCG (Fig. 3d and e). No significant differences in the levels of CAT or GSH-px (Fig. 3f and g) were observed in the colon in response to DSS with or without oral EGCG. Colonic concentration of MDA was attenuated to normal by oral EGCG (Fig. 3h). However, the effects of DSS on T-AOC, T-SOD, CAT, or MDA (Additional file 2: Figure S2d, Figure S2e, Figure S2f, and Figure S2h) were not obviously impacted by rectal EGCG. Of note, GSH-px level in the colon was significantly increased by rectal EGCG even compared with the colon of the normal group (Additional file 2: Figure S2g). However, both oral and rectal EGCG had no obvious effect on reducing apoptosis (Figs. 3i, j, S2i and S2j). To assess the effect of oral or rectal EGCG on the colonic mucosal barrier, mucin-secreting goblet cells in the colonic epithelia were measured using Alcian blue staining. DSS significantly reduced the thickness of colonic epithelial mucosa, and desirably, oral EGCG attenuated such damping to normal (Fig. 3k). Consistently, oral EGCG largely reversed DSS-induced disruption in the fine structure of the brush border and tight junctions as revealed by transmission electron microscopy (TEM) (Fig. 3l).

These results suggested that oral, but not rectal, EGCG suppressed DSS-induced oxidative stress, and attenuated DSS-damaged mucosal barrier function.

### Oral EGCG regulated the composition and SCFAs production of gut microbiota

**Fig. 4:**
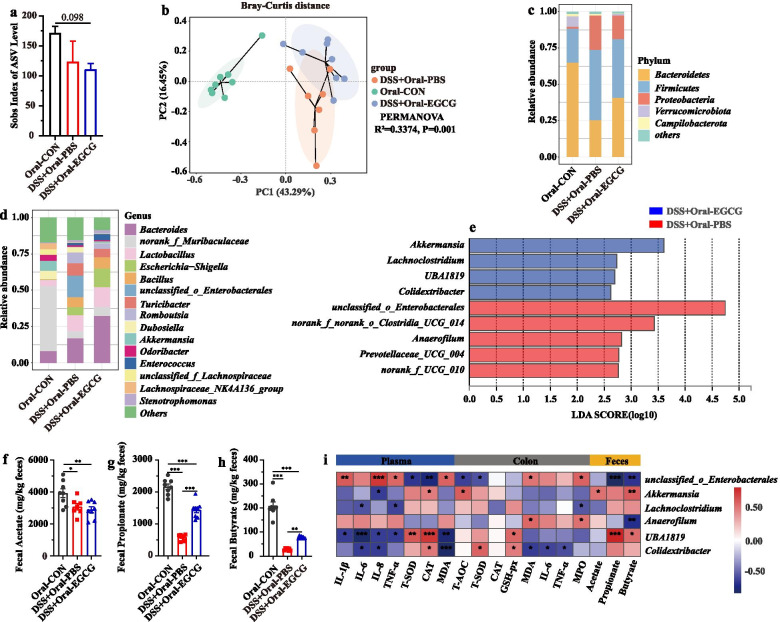
Oral EGCG regulated the composition and function of intestinal microbiota. **a** α-diversity upon oral therapy represented by the Sobs index. **b** Principal coordinate analysis (PCoA) plots upon oral therapy assessed by PERMANOVA. The relative abundance of fecal bacterial phyla (**c**); and genera (**d**) presented in 99.5% of the community upon oral therapy. **e** Analysis of differences in the microbial taxa shown by LEfSe (linear discriminant analysis (LDA) coupled with effect size measurements) upon oral therapy. Concentrations of fecal acetate (**f**), propionate (**g**), and butyrate (**h**) upon oral therapy. Data were presented as means ± SEM (*n* = 7–8 per group). Statistical significance was determined using one-way ANOVA, followed by Tukey test. *P ≤ 0.05, **P ≤ 0.01, ***P ≤ 0.001. **i** Spearman correlation between intestinal microbiota and anti-inflammatory or anti-oxidative parameters in DSS-treated mice in response to oral EGCG. The red color denotes a positive correlation, while blue color denotes a negative correlation. The intensity of the color is proportional to the strength of Spearman correlation. *P ≤ 0.05, ***P ≤ 0.001

Next, we further explore the impact of EGCG on the gut microbiota composition of DSS-treated mice receiving oral or rectal EGCG via 16S rRNA gene sequencing. Alpha diversity shown by sobs index of ASV level was impacted by neither oral EGCG nor rectal EGCG in mice with colitis (Fig. 4a, Additional file 3: Figure S3a). Principal co-ordinates analysis (PCoA) based on Bray–Curtis distance showed a separation in the gut microbiota structure among normal controls and mice with colitis (*R*^*2*^ = 0.337, *P* = 0.001, Fig. 4b; *R*^*2*^ = 0.360, *P* = 0.001, Additional file 3: Figure S3b). A separation in the gut microbiota structure was also shown between the DSS + Oral-EGCG group and DSS + Oral-PBS group (*R*^*2*^ = 0.124, *P* = 0.028), indicating that the gut microbiota structure in mice with colitis was significantly impacted by oral EGCG. Also, the fecal microbiota structure was significantly influenced by rectal EGCG (*R*^*2*^ = 0.136, *P* = 0.011).

At the phylum level, *Bacteroidetes*, *Firmicutes*, and *Proteobacteria* were predominant phyla in the fecal microbiota (Fig. 4c and Additional file 3: Figure S3c). At the genus level, the fecal microbiota was dominated by *Bacteroides*, *norank_f_Muribaculaceae*, and *Lactobacillus* (Fig. 4d and Additional file 3: Figure S3d). Differentially abundant fecal bacterial taxa in DSS-treated mice in response to oral or rectal EGCG were identified by LEfSe analysis. Additionally, we found that four bacterial genera including *Akkermansia* were enriched by oral EGCG, while the other five taxa were enriched in the DSS only group (Fig. 4e). Two bacterial genera were particularly abundant in response to rectal EGCG, while other five taxa (e.g., *Lactobacillus*, *Bacillus*, and *Romboutsia*) were enriched in the DSS only group (Additional file 3: Figure S3e).

To further explore the effect of oral or rectal EGCG on the production of SCFAs, we measured the fecal concentrations of acetate, propionate, and butyrate. All three SCFAs were significantly diminished by DSS, but the productions of propionate and butyrate were significantly improved by oral (Fig. 4f, g, and h), but not rectal EGCG (Additional file 3: Figure S3f, Figure S3g, and Figure S3h). In fact, butyrate production was further significantly reduced in DSS-treated mice in response to rectal EGCG (Additional file 3: Figure S3h).

Spearman correlation analysis was further performed to understand the association between differentially enriched microbes and anti-oxidative, inflammatory parameters, or SCFAs profiles. Correlation analysis revealed that *Akkermansia* had a strong positive correlation with the CAT level (*R* > 0.52, *P* < 0.05) in the plasma and the T-AOC level (*R* > 0.63, *P* < 0.05) in the colon and the levels of acetate (*R* > 0.56, *P* < 0.05) and butyrate (*R* > 0.65, *P* < 0.01) in the feces, but a significantly negative correlation with IL-8 (*R* <  − 0.61, *P* < 0.05) in the plasma, respectively (Fig. 4i). However, the bacteria *Lactobacillus* enriched in the colitis group had a strong negative correlation with the colonic level of TNF-α (*R* <  − 0.59, *P* < 0.05, Additional file 3: Figure S3i).

Above all, alteration of the gut microbiota and increased production of SCFAs in mice with colitis occurred in response to oral, but not rectal EGCG administration, suggested that gut microbiota might play a critical role in alleviating DSS-induced colitis.

### Prophylactic EGCG supplementation attenuated the DSS-induced colitis

**Fig. 5:**
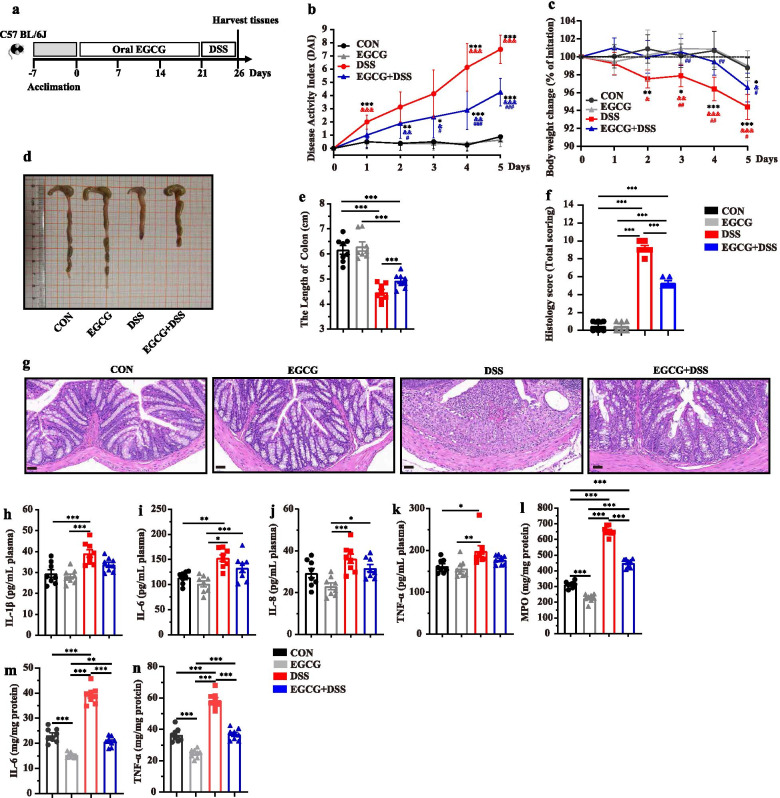
Prophylactic EGCG attenuated the symptoms of DSS-induced colitis. **a** Diagram illustrating the experimental design employed in this study. **b** Kinetics of DAI scores following DSS treatment. **c** Daily body weight changes following DSS treatment. Data were presented as means ± SEM (*n* = 8 per group). Statistical significance was determined using one-way ANOVA, followed by Tukey test. *P ≤ 0.05, **P ≤ 0.01, ***P ≤ 0.001 relative to CON group; &*P* ≤ 0.05, &&*P* ≤ 0.01, &&&*P* ≤ 0.001 relative to EGCG group; #*P* ≤ 0.05, ##*P* ≤ 0.01, ###*P* ≤ 0.001 relative to DSS group. **d** Macroscopic pictures of colons and **e** the lengths of colon from each group (*n* = 8 per group). **f** Histological scores of colons (*n* = 6 per group) and **g** H&E stained colon sections. Concentrations of four representative pro-inflammatory cytokines, IL-1β (**h**), IL-6 (**i**), IL-8 (**j**), and TNF-α (**k**) in the plasma. Concentrations of MPO (**l**), IL-6 (**m**), and TNF-α (**n**) in the colon. Data were presented as means ± SEM (*n* = 8 per group). Statistical significance was determined using one-way ANOVA, followed by Tukey test. *P ≤ 0.05, **P ≤ 0.01, ***P ≤ 0.001

Given the benefit of green tea consumption in reducing the risk of IBD [13] and different impacts on the gut microbiota by oral and rectal delivery of EGCG, prophylactic effect of EGCG on DSS-induced colitis was then explored. Mice were subjected to 2.5% DSS in drinking water for the last 6 days to induce acute colitis after receiving daily oral gavage dosed with 50 mg/kg body weight EGCG for 21 days (Fig. 5a), also as a simulation of about four to eight cups of tea per day for an adult [31]. Disease symptoms and body weight were monitored daily. Mice were then sacrificed and the colonic pathology was evaluated. The concentrations of several representative inflammatory mediators in both the plasma and the colon were also measured. Apparently, prophylactic EGCG alleviated the symptoms of colitis in DSS-treated mice as indicated by significantly reduced daily DAI (Fig. 5b) and body weight loss (Fig. 5c), and increased length of colon (Fig. 5d and e). Histological analysis further revealed that prophylactic EGCG suppressed DSS-induced infiltration of inflammatory cells and damage to the colonic mucosa (Fig. 5f and g). Moreover, prophylactic EGCG tended to reduce the plasma levels of IL-1β (Fig. 5h), IL-6 (Fig. 5i), IL-8 (Fig. 5j), and TNF-α (Fig. 5k) in mice with colitis. Strikingly, DSS-elevated levels of MPO (Fig. 5l), IL-6 (Fig. 5m), and TNF-α (Fig. 5n) in the colon were largely attenuated to normal levels by prophylactic EGCG.

These results collectively indicated that prophylactic EGCG was capable of suppressing DSS-induced colitis symptoms, colonic injury, and inflammation.

### Prophylactic EGCG reduced DSS-induced oxidative stress and apoptosis and improved the mucosal barrier function

**Fig. 6:**
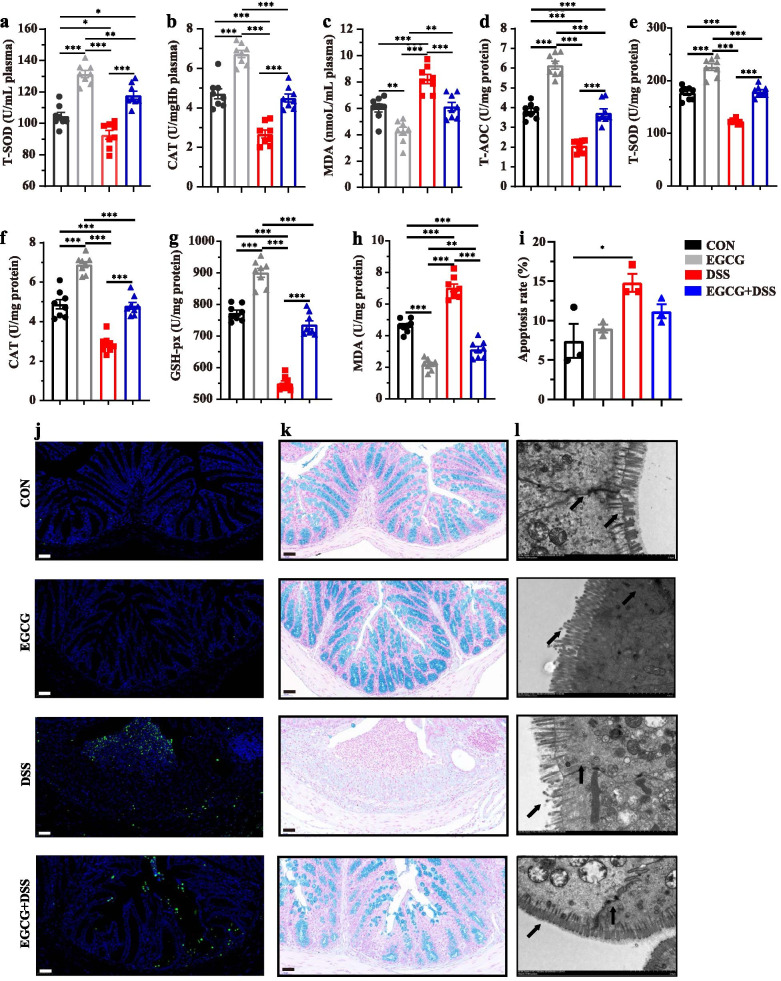
Prophylactic EGCG attenuated the oxidative stress and colonic damage. Concentrations of T-SOD (**a**), CAT (**b**), and MDA (**c**) in the plasma. Levels of T-AOC (**d**), T-SOD (**e**), CAT (**f**), GSH-px (**g**), and MDA (**h**) in the colon (*n* = 8 per group). **i** Apoptosis rate in colonic sections (*n* = 6 per group). **j** Representative fluorescent pictures of TUNEL staining of colonic sections. Scale bars represent 50 μm. **k** Representative images of Alcian blue-stained inner mucus layer of colonic sections. Scale bars represent 50 μm. **l** Representative images for the microstructure of colonic epithelia by TEM. Data were presented as Means ± SEM. Statistical significance was determined using one-way ANOVA, followed by Tukey test. *P ≤ 0.05, **P ≤ 0.01, ***P ≤ 0.001

Prophylactic EGCG largely attenuated the plasma levels of antioxidant enzymes, T-SOD, and CAT that were significantly reduced by DSS (Fig. 6a and b). Consistently, MDA in the plasma was significantly increased by DSS, but reduced to normal in response to prophylactic EGCG (Fig. 6c). Similarly, the colonic levels of T-AOC (Fig. 6d), T-SOD (Fig. 6e), CAT (Fig. 6f), and GSH-px (Fig. 6g) were significantly reduced by DSS, but were largely attenuated to the normal levels by prophylactic EGCG. The colonic level of MDA was significantly increased by DSS, but returned below normal in response to prophylactic EGCG (Fig. 6h). Of note, EGCG pre-supplementation even elicited an obvious anti-oxidative response in both the plasma and colon in healthy mice (Fig. 6a-h).

TUNEL analysis further revealed that DSS significantly increased the frequency of apoptotic cells in the colonic mucosa, but prophylactic EGCG largely reversed the trend (Fig. 6i and j). Moreover, prophylactic EGCG also attenuated the DSS-damaged colonic mucus and fine structure of colonic barrier (Fig. 6k and l).

These results suggested that prophylactic EGCG was capable of suppressing DSS-triggered oxidative stress, regulating apoptosis, and restoring mucosal barrier function in colonic epithelia.

### Prophylactic EGCG regulated the gut microbiota composition and its SCFAs production

**Fig. 7:**
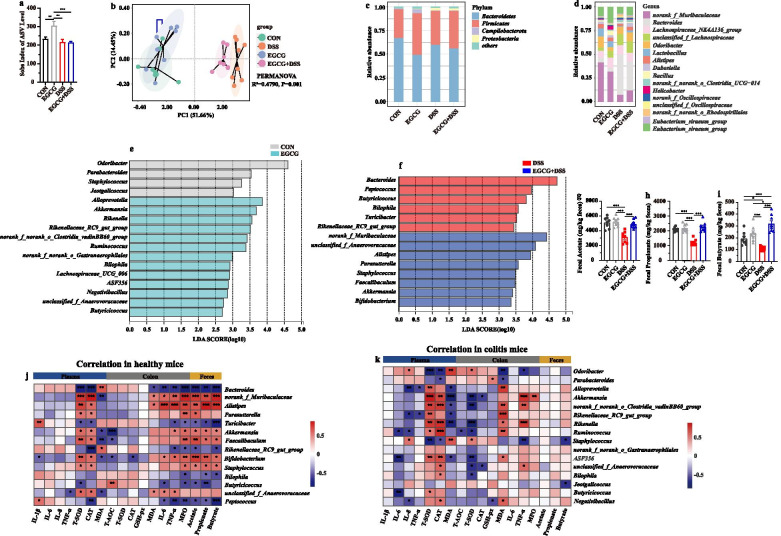
Prophylactic EGCG regulated the composition and function of intestinal microbiota. **a** α-diversity represented by the Sobs index on day 28. **b** PCoA plots on day 28 assessed by PERMANOVA among the four groups. The relative abundance of fecal bacterial phyla (**c**), and genera (**d**) presented in 99.5% of the community. Analysis of differences in the microbial taxa between the CON group and EGCG group (**e**), in DSS-treated mice with or without receiving EGCG (**f**) on day 27 was shown using LEfSe (LDA coupled with effect size measurements). Concentrations of fecal acetate (**g**), propionate (**h**), and butyrate (**i**) on day 27. Data are presented as means ± SEM (*n* = 8 per group). Statistical significance was determined using one-way ANOVA, followed by Tukey test. *P ≤ 0.05, **P ≤ 0.01, ***P ≤ 0.001. Spearman correlation between intestinal microbiota and anti-inflammatory or anti-oxidative parameters in healthy mice (**j**), and in DSS-treated mice (**k**) in response to prophylactic EGCG. The red color denotes a positive correlation, while blue color denotes a negative correlation. The intensity of the color is proportional to the strength of Spearman correlation. *P ≤ 0.05, ***P ≤ 0.001

Next, we investigated the impact of prophylactic EGCG on the gut microbiota composition of DSS-treated mice. Sobs index of ASV level was significantly increased in EGCG group compared with other three groups (*P* < 0.01, Fig. 7a). PCoA analysis showed a clear separation between control and DSS-treated groups (*R*^*2*^ = 0.479, *P* = 0.001, Fig. 7b). Additionally, prophylactic EGCG significantly affected the gut microbiota composition in between CON and EGCG group (*R*^*2*^ = 0.173, *P* = 0.001), as well as between DSS and EGCG + DSS group (*R*^*2*^ = 0.231, *P* = 0.001). Meanwhile, the overall fecal bacterial composition of mice at the phylum and genus level in all groups was consistent with the first trial (Fig. 7c and d). It was noteworthy that oral EGCG to healthy mice caused an enrichment of thirteen bacterial genera including *Akkermansia* while only four taxa were enriched in normal controls (Fig. 7e). Moreover, eight bacterial genera including *Akkermansia*, *Faecalibaculum*, and *Bifidobacterium* were enriched in mice with colitis treated by EGCG pre-supplementation, but another six genera were enriched in DSS-only group (Fig. 7f). Additionally, prophylactic EGCG completely reversed the attenuation of SCFAs including acetate (Fig. 7g), propionate (Fig. 7h), and butyrate (Fig. 7i) in DSS-treated mice.

For healthy mice, EGCG-enriched *Akkermansia* showed a strong positive correlation with the plasma levels of T-SOD (*R* > 0.84, *P* < 0.01) and CAT (*R* > 0.70, *P* < 0.05), and the colonic levels of TNF-α (*R* > 0.77, *P* < 0.01) and MPO (*R* > 0.64, *P* < 0.01), but a negative correlation with MDA level in the plasma (*R* <  − 0.79, *P* < 0.01) and the T-SOD (*R* <  − 0.71, *P* < 0.01) and CAT level (*R* <  − 0.54, *P* < 0.01) in the colon (Fig. 7j). Correlation analysis of DSS-treated mice with and without receiving prophylactic EGCG revealed that fourteen differentially bacterial genera showed significant correlation (*P* < 0.05) with the concentrations of anti-oxidative or pro-inflammatory mediator in the plasma and colon or SCFAs in the feces. Especially, *Akkermansia* was positively correlated with the plasma levels of T-SOD (*R* > 0.59, *P* < 0.05) and CAT (*R* > 0.52, *P* < 0.05), the colonic level of TNF-α (*R* > 0.52, *P* < 0.01) and MPO (*R* > 0.59, *P* < 0.05), and the fecal production of acetate (*R* > 0.55, *P* < 0.05), propionate (*R* > 0.55, *P* < 0.05), and butyrate (*R* > 0.50, *P* < 0.05). Negative associations with the plasma level of MDA (*R* <  − 0.60, *P* < 0.05) and colonic level of T-AOC (*R* <  − 0.81, *P* < 0.01) were also shown (Fig. 7k).

Above all, these results indicated that prophylactic EGCG regulated the gut microbiota composition and its metabolism, leading to the potential to attenuate the DSS-induced dysbiosis.

### EGCG-FMT contributed more to alleviating colitis than CON-FMT and EGCG-SFF

**Fig. 8:**
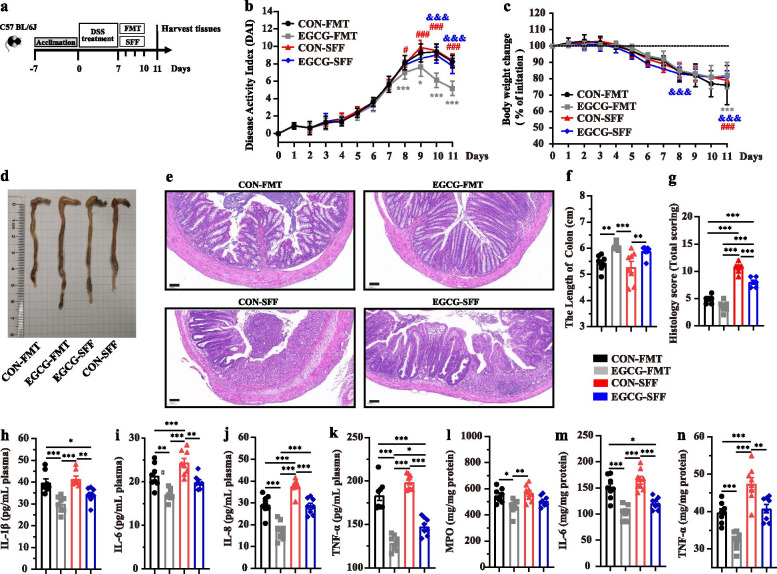
EGCG-FMT alleviated DSS-induced experimental colitis better than CON-FMT and EGCG-SFF. **a** Diagram illustrating the mouse model of colitis employed in this study. EGCG-FMT and CON-FMT treatments were indicated. **b** Kinetics of DAI scores throughout the entire duration of the study. **c** Daily body weight changes throughout the entire duration of the study. **d** Macroscopic pictures of colons and (**f**) the lengths of colon from each group. **E** H&E stained colon sections and (**g**) histological scores of colons (*n* = 6 per group). Concentrations of four representative pro-inflammatory cytokines, IL-1β (**h**), IL-6 (**i**), IL-8 (**j**), and TNF-α (**k**) in the plasma. Concentrations of MPO (L), IL-6 (M), and TNF-α (N) in the colon. Data were presented as means ± SEM (*n* = 8 per group). Statistical significance was determined using one-way ANOVA, followed by Tukey test. *P ≤ 0.05, **P ≤ 0.01, ***P ≤ 0.001

We next validated the impact of EGCG-mediated microbiota on murine colitis in the third animal trial via transplanting the fecal microbiota derived from mice receiving EGCG gavage to DSS-treated mice (Fig. 8a). As before, more increased body weight and colon length, and decreased DAI and histological damage was shown in mice with colitis treated by EGCG-FMT compared with other three groups (Fig. 8b-g). As well, decreased levels of IL-1β, IL-6, IL-8, and TNF-α in the plasma (Fig. 8h-k), and decreased levels of IL-6 and TNF-α, and increased level of MPO (Fig. 8l-n) in the colon were shown in mice with colitis treated by EGCG-FMT compared with other three groups. Notably, it could not be neglected that EGCG-SFF also more profoundly alleviated the symptoms of acute colitis compared to mice with CON-SFF, indicated by less body weight loss, DAI score, and histological score, increased colonic length (Fig. 8b-g). Also, the plasma levels of IL-1β, IL-6, IL-8 (Fig. 8h-k), and TNF-α, and the colonic levels of IL-6 and TNF-α (Fig. 8h, m) were decreased by EGCG-SFF compared with that of CON-SFF.

### EGCG-FMT contributed more to alleviating the colonic barrier damage than CON-FMT and EGCG-SFF

**Fig. 9:**
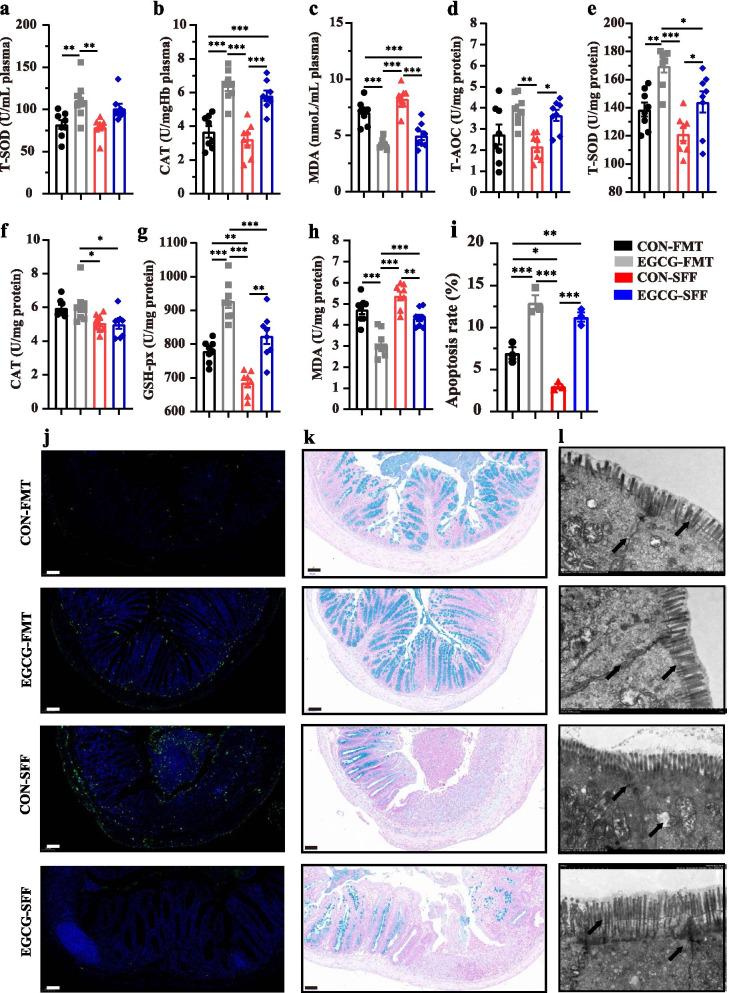
EGCG-FMT alleviated oxidative stress and colonic damage better than CON-FMT and EGCG-SFF. Concentrations of T-SOD (**a**), CAT (**b**), and MDA (**c**) in the plasma from each group. Levels of T-AOC (**d**), T-SOD (**e**), CAT (**f**), GSH-px (**g**), and MDA (**h**) in the colon (*n* = 8 per group). **i** Apoptosis rate in colonic sections (*n* = 6 per group). **j** Representative fluorescent pictures of TUNEL staining of colonic sections. Scale bars represent 50 μm. **k** Representative images of Alcian blue-stained inner mucus layer of colonic sections. Scale bars represent 50 μm. **l** Representative images for the microstructure of colonic epithelia by TEM. Data were presented as means ± SEM. Statistical significance was determined using one-way ANOVA, followed by Tukey test. *P ≤ 0.05, **P ≤ 0.01, ***P ≤ 0.001

Increased concentrations of T-SOD and CAT, and decreased concentration of MDA in the plasma of mice with colitis were observed by EGCG-FMT compared with other three groups (Fig. 9a-c). Also, increased concentrations of T-SOD and GSH-PX, and decreased concentration of MDA in the colon of mice with colitis were shown by EGCG-FMT compared with other three groups (Fig. 9e-h). Meanwhile, microbiota from EGCG-dosed mice promoted cell apoptosis and maintained better mucus distribution and integrity of tight junction in the colon in mice with colitis compared with CON-FMT or EGCG-SFF (Fig. 9i-l). Notably, similar differences were shown in mice with EGCG-SFF compared to CON-SFF, indicated by increased plasma level of CAT, and colonic levels of T-SOD and GSH-PX, and decreased level of MDA in the plasma and colon (Fig. 9a-h). And the EGCG-SFF also promoted the apoptosis of colonic epithelium (Fig. 9i and j).

### EGCG-FMT promoted the enrichment of SCFA-producing bacteria and subsequent production of SCFAs

**Fig. 10:**
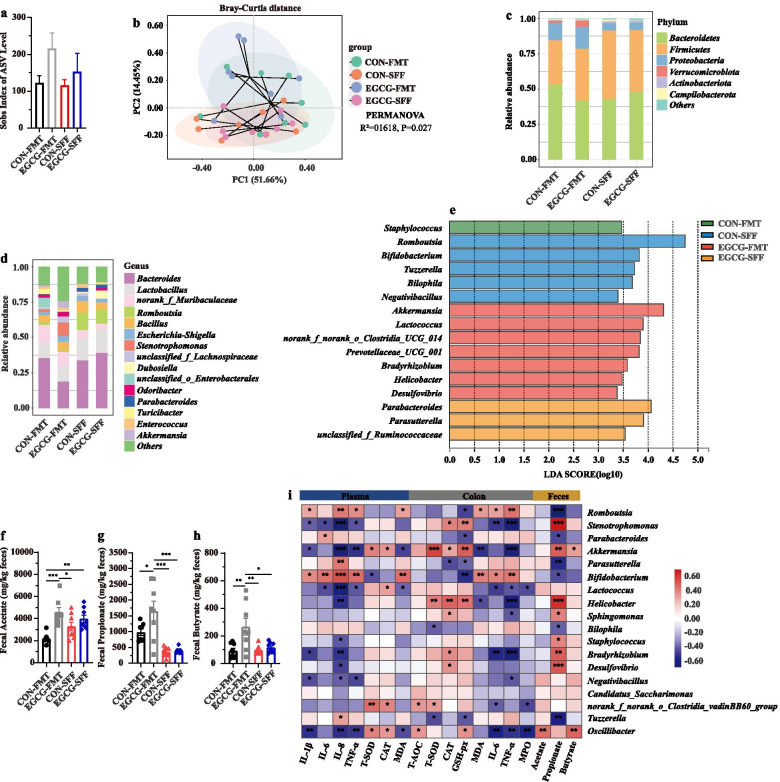
EGCG-FMT promoted the enrichment of SCFAs-producing bacteria and production of SCFAs. **a** α-diversity upon oral therapy represented by the Sobs index. **b** PCoA plots upon FMT or SFF assessed by PERMANOVA analysis. The relative abundance of fecal bacterial phyla (**c**), and genera (**d**) presented in 99.5% of the community. **e** Analysis of differences in the microbial taxa shown by LEfSe (LDA coupled with effect size measurements) upon FMT. Concentrations of fecal acetate (**f**), propionate (**g**), and butyrate (**h**) upon FMT. Data were presented as means ± SEM (*n* = 8 per group). Statistical significance was determined using one-way ANOVA, followed by Tukey test. *P ≤ 0.05, ***P ≤ 0.001. **i** Spearman correlation between intestinal microbiota and anti-inflammatory or anti-oxidative parameters in DSS-treated mice in response to FMT or SFF. The red color denotes a positive correlation, while blue color denotes a negative correlation. The intensity of the color is proportional to the strength of Spearman correlation. *P ≤ 0.05, ***P ≤ 0.001

The composition of gut microbiota upon FMT or SFF was also analyzed. Sobs index of ASV level was not significantly impacted by FMT or SFF in mice with colitis (Fig. 10a). PCoA analysis revealed the change of structure among all the four groups (*R*^*2*^ = 0.169, *P* = 0.003, Fig. 10b), between two groups with FMT (*R*^*2*^ = 0.094, *P* = 0.099), between two groups with SFF (*R*^*2*^ = 0.065, *P* = 0.406), as well as between mice with CON-FMT and CON-SFF (*R*^*2*^ = 0.148, *P* = 0.015). Especially, it revealed significantly separated structure between mice with EGCG-FMT and EGCG-SFF (*R*^*2*^ = 0.226, *P* = 0.001). The main composition in both phylum and genus levels was similar among groups (Fig. 10c and d). Of note, the genus *Akkermansia*, was significantly enriched by EGCG-FMT (Fig. 10e). Also, the productions of fecal SCFAs (acetate, propionate, and butyrate) in mice with colitis were significantly increased by EGCG-FMT than other three groups where the productions of SCFAs were not significantly impacted by EGCG-SFF compared to CON-SFF(Fig. 10f, g, and h). Moreover, correlation analysis revealed that differentia microbe *Akkermansia* was positively correlated with the plasma levels of T-SOD (*R* > 0.44, *P* < 0.05) and CAT (*R* > 0.39, *P* < 0.05), the colonic levels of T-SOD (*R* > 0.62, *P* < 0.01), CAT (*R* > 0.35, *P* < 0.05), and GSH-px (*R* > 0.54, *P* < 0.01), and the fecal production of propionate (*R* > 0.53, *P* < 0.01) and butyrate (*R* > 0.35, *P* < 0.05). Negative associations with the plasma levels of IL-1β (*R* <  − 0.38, *P* < 0.05), IL-8 (*R* <  − 0.58, *P* < 0.01), TNF-α (*R* <  − 0.47, *P* < 0.01), and MDA (*R* <  − 0.42, *P* < 0.05), and colonic levels of MDA (*R* <  − 0.46, *P* < 0.01), and TNF-α (*R* <  − 0.60, *P* < 0.01) were also shown (Fig. 10i).

Overall, EGCG-FMT alleviated colitis and colonic barrier damage in mice with colitis more profoundly than CON-FMT or EGCG-SFF. In agreement with our therapeutic and pre-supplemented trials, enriched *Akkermansia* and increased SCFAs production were also induced by EGCG-FMT, which indicated alleviated effects of gut microbiota from EGCG-dosed mice on colitis.

## Discussion

Besides host genetics, environmental factors such as dietary preference and gut microbiota have been associated with the development and progression of IBD [11, 13, 32–34]. Gut microbiota constitutes a critical bridge between environmental factors and host health, where the metabolites of microbiota such as SCFAs may exert anti-inflammatory function [35–37]. Daily consumption of tea has been shown to reduce the risk of IBD [12, 13], and whether the protective effects of its functional components associate with gut microbiota has arisen the attention [38, 39]. In agreement with our results between normal controls and mice with colitis, gut microbiota dysbiosis is the character of intestinal diseases, where the therapeutic strategies in modulating gut microbiota such as FMT have arisen the attention [40, 41]. Here, we found that EGCG modulated gut microbiota composition and its metabolites, namely, enriching the SCFAs-producing bacteria such as *Akkermansia*, increasing the SCFAs production, and therefore promoting an anti-inflammatory and anti-oxidative state in the gut. Also, we demonstrate that gut microbiota and its metabolites induced by EGCG played a key role in the attenuation of DSS-induced experimental colitis, as supported by the more profoundly alleviated effects from EGCG-FMT than CON-FMT and EGCG-SFF.

To explore how oral or rectal EGCG attenuated the colitis, acute colitis of mice was induced by 2.5% DSS and then mice were treated by oral or rectal EGCG. In agreement with previous studies [16, 17], we confirmed that the therapeutic applications of oral EGCG have led to the alleviation in DSS-induced colitis, as indicated by the decreased DAI and histological damage. Consistent with previous studies [16, 42–45], oral EGCG also alleviated colonic inflammation as exemplified by the decline of inflammatory cytokines such as IL-6 and TNF-α in both the plasma and colon of DSS-treated mice. Furthermore, we found that rectal delivery of EGCG failed to attenuate intestinal inflammation and pathology, which was supported by its little toxicity on rectal mucosa [46]. Moreover, therapeutic oral EGCG significantly attenuated DSS-induced oxidative stress by increasing the synthesis of antioxidant enzymes such as T-SOD and CAT, and decreasing the production of MDA in the colon and plasma. In agreement with it, a previous study has revealed that therapeutic EGCG showed similar anti-oxidative effects [17]. Moreover, oral EGCG alleviated DSS-induced damage in integrity and barrier function as evidenced by the increased colonic mucus as well as the enhanced tight junctions and ultrastructure of the colonic mucosa in agreement with previous studies [47–50] while rectal EGCG did not. Above all, different exposure of EGCG resulted in reversed effects, namely, oral EGCG alleviated colitis while rectal EGCG even exacerbated it. Considering that rectal EGCG has previously been demonstrated to impact the rectal infection of viruses [46], we hypothesized that those reversed results were due to their different impacts on gut microbiota and its metabolism.

Importantly, oral and rectal EGCG differentially impacted the gut microbiota and its metabolism. PCoA analyses revealed that gut microbiota structure was significantly impacted by oral EGCG and rectal EGCG, though community richness revealed by sobs index was not obviously impacted in mice with colitis by these two delivery routes. As expected, therapeutic oral EGCG significantly increased SCFAs-producing bacteria including *Akkermansia*, and subsequently promoted the production of SCFAs. Moreover, the correlation analysis suggested that the relative abundance of *Akkermansia* was positively correlated with the SCFAs production, anti-oxidant enzymes, but negatively correlated with pro-inflammatory cytokines. On the other hand, *Akkermansia* was not enriched by rectal EGCG. Similarly, previous studies have revealed that EGCG effectively reduced diet-increased obesity, and promoted the colonization of *Akkermansia muciniphila*, but how it impacted microbiota metabolites was unclear [38, 51]. Also, a recent study has shown that the high fat diet-induced mRNA expression levels of pro-inflammatory cytokines IL-6, IL-1β were significantly reversed by *Akkermansia muciniphila* [52]. However, *Lactobacillus* levels and butyrate production in mice with colitis were further decreased by rectal EGCG compared to the group treated only with DSS. *Lactobacillus*, as the most common probiotics, is considered to exert extensive anti-inflammatory effects where lactate may play a role [53]. The decreased *Lactobacillus* levels and butyrate production have been demonstrated to induce the exacerbation of colitis in mice [11]. These outcomes indicated that the composition and metabolites of gut microbiota in mice with colitis were differentially modulated by the two exposure ways of EGCG. Oral EGCG contributed to a stable microenvironment while rectal EGCG reversely. Also, the altered gut microbiota and its metabolites derived from EGCG intervention might play a central role in these reversals.

Next, we further validated these probiotic effects by long-term administration of prophylactic EGCG, also as a simulation of about four to eight cups of tea per day for an adult [31]. The mechanism of this exposure route on attenuating the experimental colitis was also explored. As with therapeutic oral EGCG, prophylactic EGCG effectively attenuated the symptoms of colitis. Prophylactic EGCG also declined the levels of pro-inflammatory cytokines and increased the anti-oxidative level in both the plasma and colon of DSS-treated mice. Furthermore, prophylactic EGCG was also capable of increasing the level of GSH-px in the colon which might promote the anti-oxidative state more profoundly. Also, integrity and barrier function was protected from DSS-induced damage by prophylactic EGCG. Interestingly, prophylactic EGCG obviously decreased the pro-inflammatory state and promoted anti-oxidative state of intestine in healthy mice, indicating that consumption of EGCG had the potential to improve intestinal barrier.

The microbiota composition and its metabolites were also significantly modulated by prophylactic EGCG in both healthy mice and mice with colitis. Community richness was not obviously impacted in mice with colitis by prophylactic EGCG. Interestingly, we found that prophylactic EGCG significantly increased the community richness in healthy mice, suggesting the potentially beneficial effects of EGCG in gut microenvironment for animals or humans in the future. Also, the structure of microbial community was significantly impacted by prophylactic EGCG in both healthy mice and mice with colitis, reminding us that the altered microbiota might play a vital role in alleviating the development of colitis. Moreover, prophylactic EGCG significantly increased SCFAs-producing bacteria including *Akkermansia*, and subsequently promoted the production of SCFAs, which was in accordance with therapeutic oral EGCG. Similarly, the SCFAs-producing bacteria *Akkermansia* positively correlated with SCFAs production and anti-oxidant enzymes, but negative correlated with pro-inflammatory cytokines of mice upon prophylactic EGCG. And potential probiotics *Bifidobacterium* and *Faecalibaculum* were also enriched in mice with colitis treated by prophylactic EGCG. Previous researches showed that *Akkermansia muciniphila* could release two kinds of SCFAs (acetate and propionate), and the cross-feeding between *Akkermansia muciniphila* and butyrate-producing bacteria promoted the butyrate production [54]. Also, this similar cross-feeding has been shown between *Bifidobacterium* and butyrate-producing bacteria [55]. The endogenous *Faecalibaculum rodentium* in the murine gut has been shown to promote intestinal tumorigenesis where SCFAs plays a vital role [56]. These results indicated that SCFAs-producing bacteria and subsequent production of SCFAs might play a role in the attenuation of colitis.

We next sought to demonstrate the role of SCFAs-producing bacteria and subsequent production of SCFAs mediated by EGCG and verified whether the impacted gut microbiota and metabolites was the results or the reason of alleviated colitis. The murine model with experimental colitis was induced, and FMT or SFF from normal controls or EGCG-dosed mice were performed. Interestingly, acute colitis was successfully alleviated by EGCG-FMT. In consistent with that of EGCG itself, EGCG-FMT alleviated symptoms of colitis profoundly better than CON-FMT, suggesting that EGCG-mediated gut microbiota plays a dominant role in alleviating the colitis. Also, EGCG-SFF showed better alleviated symptoms than CON-SFF, suggesting the role of metabolites could not be neglected. EGCG-FMT significantly attenuated DSS-induced inflammatory response and oxidative stress shown by similar performances with those of oral EGCG and prophylactic EGCG. Based on those results, we demonstrated that gut microbiota and its metabolites mediated by EGCG was the reason for the alleviated effects. Combining the significant impacts of prophylactic EGCG on microbial community in healthy mice and the successfully alleviated effects from EGCG-FMT, we believed that EGCG facilitated the other microbes which were of low abundance in normal controls and hence ameliorates colitis.

We then explored the difference between EGCG-FMT and EGCG-SFF. FMT and SFF did not significantly impact the community richness. The structure of microbial community was significantly impacted by EGCG-FMT while EGCG-SFF did not, which might because of the sterility in SFF. As expected, EGCG-FMT significantly increased SCFAs-producing bacteria such as *Akkermansia*, and subsequently promoted the production of SCFAs. *Akkermansia* has also shown a similar correlation with the inflammatory and oxidative indexes from oral therapy of EGCG and prophylactic EGCG. These outcomes indicated that the alleviated effect of EGCG-FMT was similar with that of EGCG itself. Notably, EGCG-FMT significantly promoted the production of SCFAs while EGCG-SFF did not, indicating that the release of SCFAs depended on SCFAs-producing bacteria. Based on our results and the previous studies, the SCFAs and SCFAs-producing microbes *Akkermansia* might also exert a beneficial role in the interplay between the gut microbiota and green tea polyphenols for the therapy or prevention of diseases [57–60].

Even though it has been previously shown that EGCG directly upregulate anti-oxidative function and impact cell proliferation in vitro [18, 19]. We thought that these direct effects did not play a primary role in vivo, as indicated by our results that mice with colitis treated by rectal EGCG trended to exacerbate colitis. As the most abundant tea polyphenol in green tea, it was likely that the beneficial effects of EGCG were mediated through more than one mechanism [61, 62]. Besides the functional SCFAs, previous studies have revealed that some phenolic metabolites from EGCG degradation may play a critical role, alongside other beneficial activities of EGCG, in reducing intestinal inflammatory diseases [63, 64]. It cannot be denied that mice treated by oral EGCG or prophylactic EGCG benefited from not only gut microbiota and functional SCFAs but also other phenolic metabolites. Based on our results, we believe that these functional secondary metabolites of EGCG did not play a major role in the alleviated symptoms. This is mainly due to the fact that the major phenolic metabolites of EGCG such as phenyl-γ-valerolactones are easily absorbed in the body after degradation of EGCG by intestinal bacteria and excreted via urine [14, 63]. In addition, EGCG has been metabolized and absorbed in donors when we collected feces for FMT and SFF, indicating that the fecal concentrations of these metabolites were pretty low. However, the functional SCFAs could be continuously produced by SCFAs-producing bacteria depending on basal carbon and nitrogen source. Probably due to this, EGCG-SFF has also showed anti-inflammatory and anti-oxidative effects compared to CON-SFF, which seemed to be similar to EGCG-FMT. Moreover, EGCG-FMT alleviated the acute colitis better than CON-FMT and SFF, suggesting that EGCG-modulated gut microbiota played a vital role in alleviating colitis. Furthermore, EGCG-FMT enriched the SCFAs-producing bacteria such as *Akkermansia* and further increased the fecal level of SCFAs compared with EGCG-SFF, indicating that EGCG-FMT exerted more beneficial effects in the colitis of mice. Based on this, we believe that the anti-inflammatory and anti-oxidative effects of SCFA-producing bacteria enriched by EGCG were mainly mediated by functional SCFAs. In addition, the extra addition of gut microbiota further contributes to subsequent production of SCFAs than SFF.

**Fig. 11:**
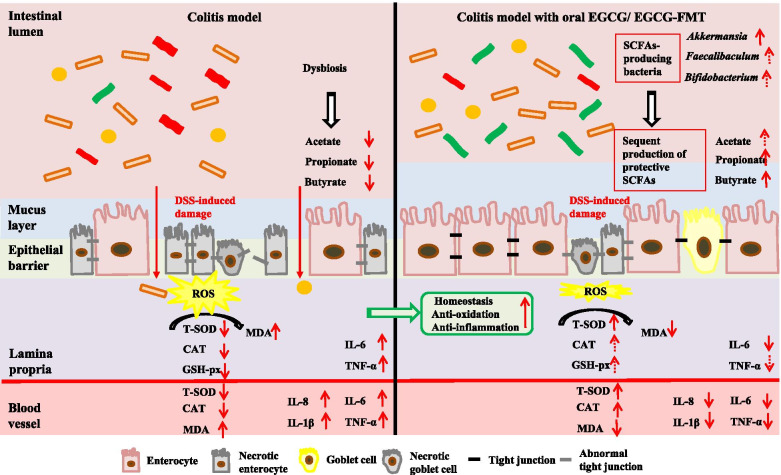
A schematic model showing the mechanism by which oral, but not rectal, delivery of EGCG alleviated DSS-induced colitis. Intestinal microbiota, oxidative stress, inflammation, and barrier integrity are all affected. Oral EGCG induced an alteration in the gut microbiota to enrich probiotic bacteria such as *Akkermansia*, which subsequently led to an increased production of SCFAs such as butyrate, triggering a cascade of anti-oxidative, anti-inflammatory, and barrier-protective response. Ultimately, intestinal epithelial homeostasis is attenuated and colitis was attenuated

Collectively, our results suggested that EGCG-mediated microbial community, especially the enrichment of *Akkermansia* played a key role in the alleviation of colitis, which could be the major driving force behind reversal outcomes between oral and rectal deliveries of EGCG. These increased populations of SCFAs-producing microbes and their metabolites induced by EGCG delivery might also be involved in maintaining the homeostatic balance in the colon by improving the colonic epithelial integrity and mucosal immunity [65, 66]. These results are summarized in Fig. 11. Taken together, we concluded that SCFAs-producing bacteria induced by EGCG, especially such as *Akkermansia*, and subsequent production of protective SCFAs contributes to the anti-oxidative and anti-inflammatory state, and further protected from the damage in colon.

## Conclusions

In summary, oral, but not rectal, delivery of EGCG attenuates DSS-induced colitis, and their different impacts on the composition and metabolites of gut microbiota, implies a central role of gut microbiota in mediating the beneficial effects. Given the alleviated effect of microbiota from EGCG-dosed mice, the beneficial role for alleviating IBD progress by EGCG was believed to be mediated mainly through the SCFAs-producing bacteria especially such as *Akkermansia* and subsequent production of functional SCFAs. These changes in gut microbiota subsequently lead to increased production of protective SCFAs such as butyrate, which in turn triggers a pronounced anti-oxidative, anti-inflammatory, and barrier-enhancing program, resulting in the attenuation of intestinal inflammation and damage. The proposed mechanisms are summarized in Fig. 11. These findings provide novel insights into the EGCG-mediated alleviation of IBD and will facilitate the development of therapeutic and preventive strategies for IBD and other inflammatory disorders.

## Supplementary Information

**Additional file 1: Figure S1.** Rectal EGCG had a minimum effect on alleviating DSS-induced colitis. (**a**) Diagram illustrating the mouse model of colitis employed in this study. Rectal PBS and EGCG treatments were indicated. (**b**) Kinetics of DAI scores throughout the entire duration of the study. (**c**) Daily body weight changes throughout the entire duration of the study. Data were presented as Means ± SEM (*n* = 7 per group). Statistical significance was determined using one-way ANOVA, followed by Turkey test. ** *P* ≤ 0.01, *** *P* ≤ 0.001 relative to Rectal-CON group; ## *P* ≤ 0.01, ### *P* ≤ 0.001 relative to DSS + Rectal-PBS group. (**d**) Macroscopic pictures of colons and (**e**) the lengths of colon from each group (*n* = 7 per group). (**f**) Histological scores of colons (*n *= 6 per group) and (**g**) H&E stained colon sections. Concentrations of four representative pro-inflammatory cytokines, IL-1β (**h**), IL-6 (**i**), IL-8 (**j**), and TNF-α (**k**) in the plasma. Concentrations of MPO (**l**), IL-6 (**m**), and TNF-α (**n**) in the colon. Data were presented as Means ± SEM (*n* = 7 per group). Statistical significance was determined using one-way ANOVA, followed by Turkey test. * *P* ≤ 0.05, ** *P* ≤ 0.01, *** *P* ≤ 0.001.

**Additional file 2: Figure S2.** Rectal EGCG played a minimum role in the oxidative stress and colonic damage. Concentrations of T-SOD (**a**), CAT (**b**), and MDA (**c**) in the plasma from each group. Levels of T-AOC (**d**), T-SOD (**e**), CAT (**f**), GSH-px (**g**), and MDA (**h**) in the colon. Data were presented as Means ± SEM (*n* = 7 per group). (**i**) Apoptosis rate in colonic sections (*n* = 6 per group). (**j**) Representative fluorescent pictures of TUNEL staining of colonic sections. Scale bars represent 50 μm. (**k**) Representative images of Alcian blue stained inner mucus layer of colonic sections. Scale bars represent 50 μm. (**l**) Representative images for the microstructure of colonic epithelia by TEM. Data were presented as Means ± SEM. Statistical significance was determined using one-way ANOVA, followed by Turkey test. * *P* ≤ 0.05, ** *P* ≤ 0.01, *** *P* ≤ 0.001.

**Additional file 3: Figure S3.** Oral EGCG regulated the composition and function of intestinal microbiota. (**a**) α-diversity upon oral therapy represented by the Sobs index. (**b**) PCoA plots upon rectal therapy assessed by PERMANOVA. The relative abundance of fecal bacterial phyla (**c**), and genera (**d**) presented in 99.5% of the community upon rectal therapy. (**e**) Analysis of differences in the microbial taxa shown by LEfSe (LDA coupled with effect size measurements) upon rectal therapy. Concentrations of fecal acetate (**f**), propionate (**g**), and butyrate (**h**) upon rectal therapy. Data were presented as Means ± SEM (*n* = 7 per group). Statistical significance was determined using one-way ANOVA, followed by Turkey test. * *P *≤ 0.05, ** *P* ≤ 0.01, *** *P* ≤ 0.001. (**i**) Spearman Correlation between intestinal microbiota and anti-inflammatory or anti-oxidative parameters in DSS-treated mice in response to rectal EGCG. The red color denotes a positive correlation, while blue color denotes a negative correlation. The intensity of the color is proportional to the strength of Spearman correlation. * *P *≤ 0.05, *** *P* ≤ 0.001.

**Additional file 4.** Full account of statistical analysis performed in R software (version 3.3.1).

## Data Availability

The datasets supporting the conclusions of this article are available in the NCBI Sequence Read Archive (SRA) repository under accession number PRJNA613584 and PRJNA679405 (available on February 01, 2021).

## Abbreviations

IBD: : Inflammatory bowel disease
EGCG: Epigallocatechin-3-gallate
DSS: Dextran sulfate sodium
SCFA: Short-chain fatty acid
FMT: Fecal microbiota transplantation
SPF: Specific pathogen-free
DAI: Disease activity index
SFF: Sterile fecal filtrate
TUNEL: Terminal deoxynucleotidyl transferase-mediated dUTP nick end labeling
TEM: Transmission electron microscopy
KEGG: Kyoto Encyclopedia of Genes and Genomes
LDA: Linear discriminant analysis
MPO: Myeloperoxidase
T-AOC: Total antioxidant capacity
T-SOD: Total superoxide dismutases
CAT: Catalase
GSH-px: Glutathione peroxidase
MDA: Malondialdehyde
PCoA: Principal co-ordinates analysis
