# Supplementary figures and images for: Gut microbiota from green tea polyphenol-dosed mice improves intestinal epithelial homeostasis and ameliorates experimental colitis
Source: Microbiome. 2021 Sep 7;9:184. doi: 10.1186/s40168-021-01115-9 (PMC8424887; doi:10.1186/s40168-021-01115-9)

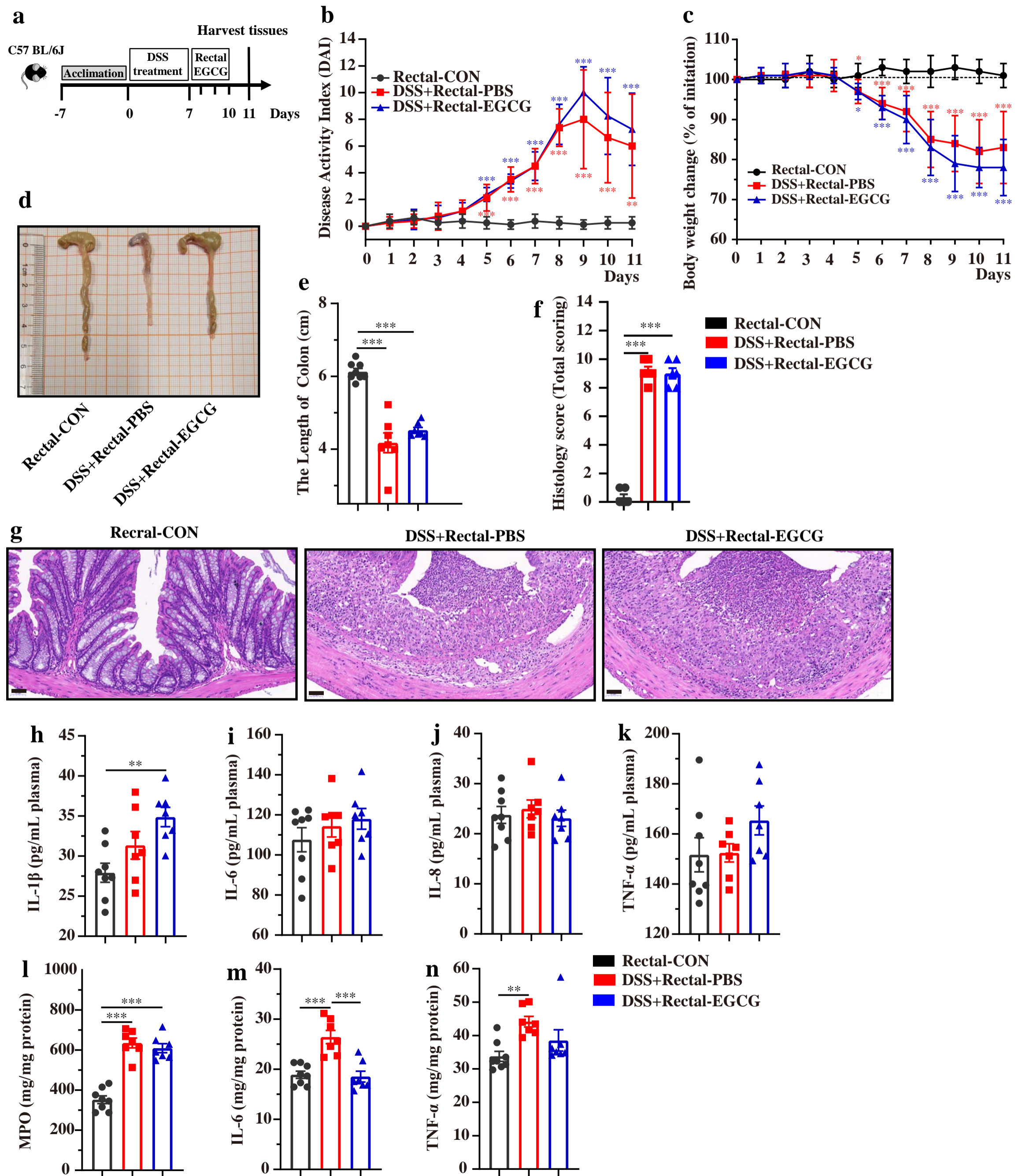

Supplement: Supplementary file 2 — Additional file 1: Figure S1. Rectal EGCG had a minimum effect on alleviating DSS-induced colitis. (a) Diagram illustrating the mouse model of colitis employed in this study. Rectal PBS and EGCG treatments were indicated. (b) Kinetics of DAI scores throughout the entire duration of the study. (c) Daily body weight changes throughout the entire duration of the study. Data were presented as Means ± SEM (n = 7 per group). Statistical significance was determined using one-way ANOVA, followed by Turkey test. ** P ≤ 0.01, *** P ≤ 0.001 relative to Rectal-CON group; ## P ≤ 0.01, ### P ≤ 0.001 relative to DSS + Rectal-PBS group. (d) Macroscopic pictures of colons and (e) the lengths of colon from each group (n = 7 per group). (f) Histological scores of colons (n = 6 per group) and (g) H&E stained colon sections. Concentrations of four representative pro-inflammatory cytokines, IL-1β (h), IL-6 (i), IL-8 (j), and TNF-α (k) in the plasma. Concentrations of MPO (l), IL-6 (m), and TNF-α (n) in the colon. Data were presented as Means ± SEM (n = 7 per group). Statistical significance was determined using one-way ANOVA, followed by Turkey test. * P ≤ 0.05, ** P ≤ 0.01, *** P ≤ 0.001. [file 40168_2021_1115_MOESM1_ESM.pdf]

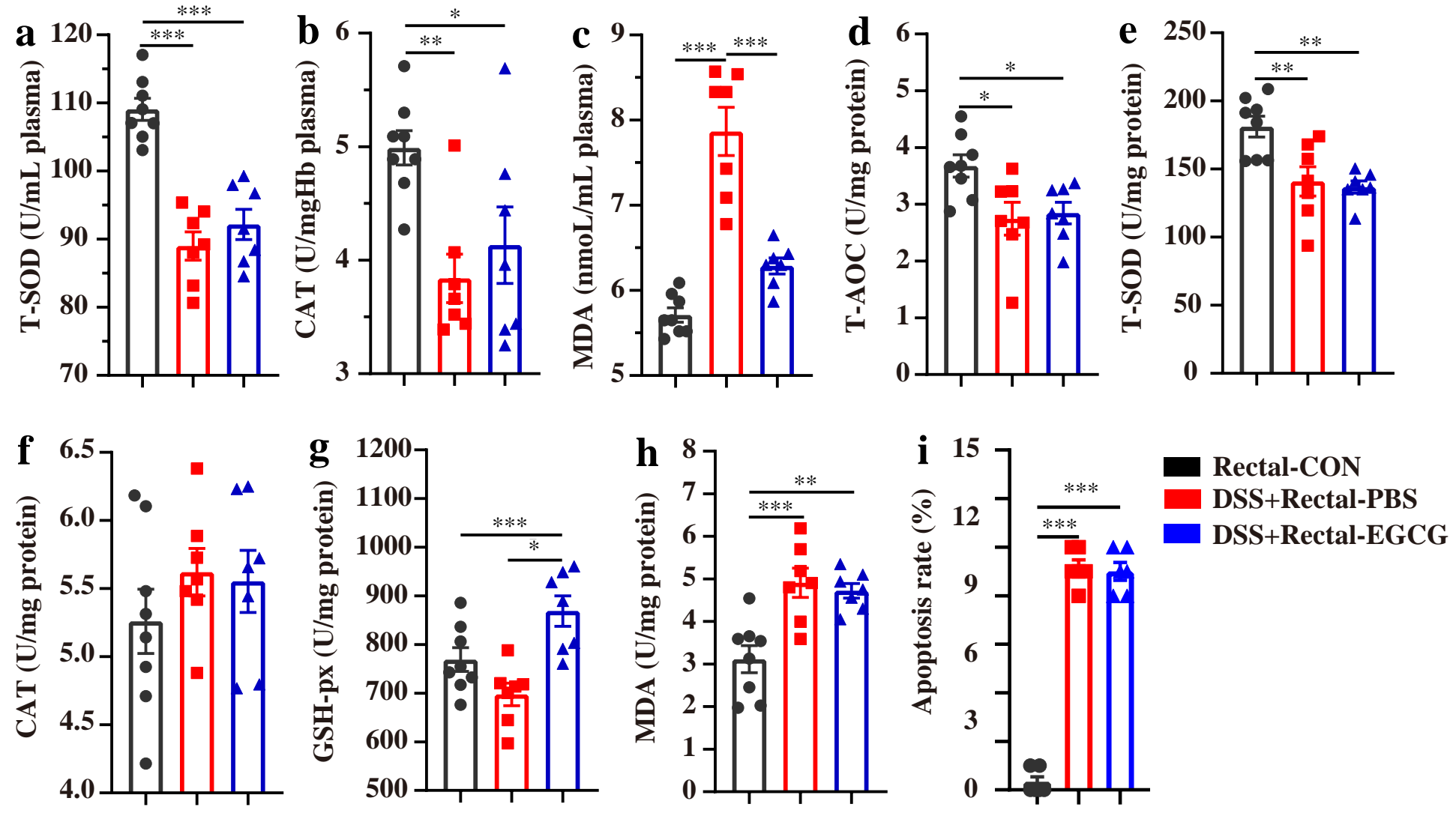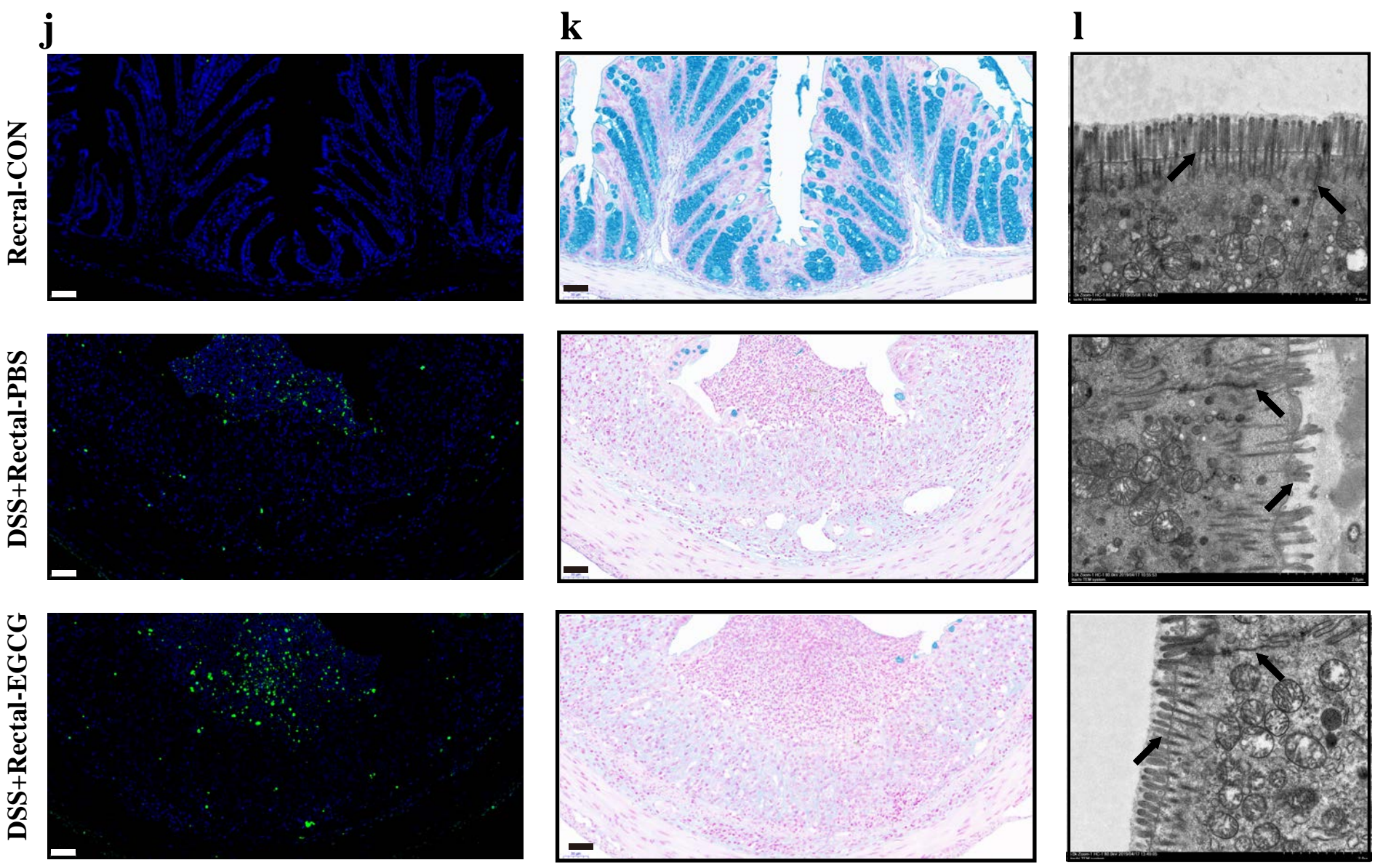

Supplement: Supplementary file 3 — Additional file 2: Figure S2. Rectal EGCG played a minimum role in the oxidative stress and colonic damage. Concentrations of T-SOD (a), CAT (b), and MDA (c) in the plasma from each group. Levels of T-AOC (d), T-SOD (e), CAT (f), GSH-px (g), and MDA (h) in the colon. Data were presented as Means ± SEM (n = 7 per group). (i) Apoptosis rate in colonic sections (n = 6 per group). (j) Representative fluorescent pictures of TUNEL staining of colonic sections. Scale bars represent 50 μm. (k) Representative images of Alcian blue stained inner mucus layer of colonic sections. Scale bars represent 50 μm. (l) Representative images for the microstructure of colonic epithelia by TEM. Data were presented as Means ± SEM. Statistical significance was determined using one-way ANOVA, followed by Turkey test. * P ≤ 0.05, ** P ≤ 0.01, *** P ≤ 0.001. [file 40168_2021_1115_MOESM2_ESM.pdf]

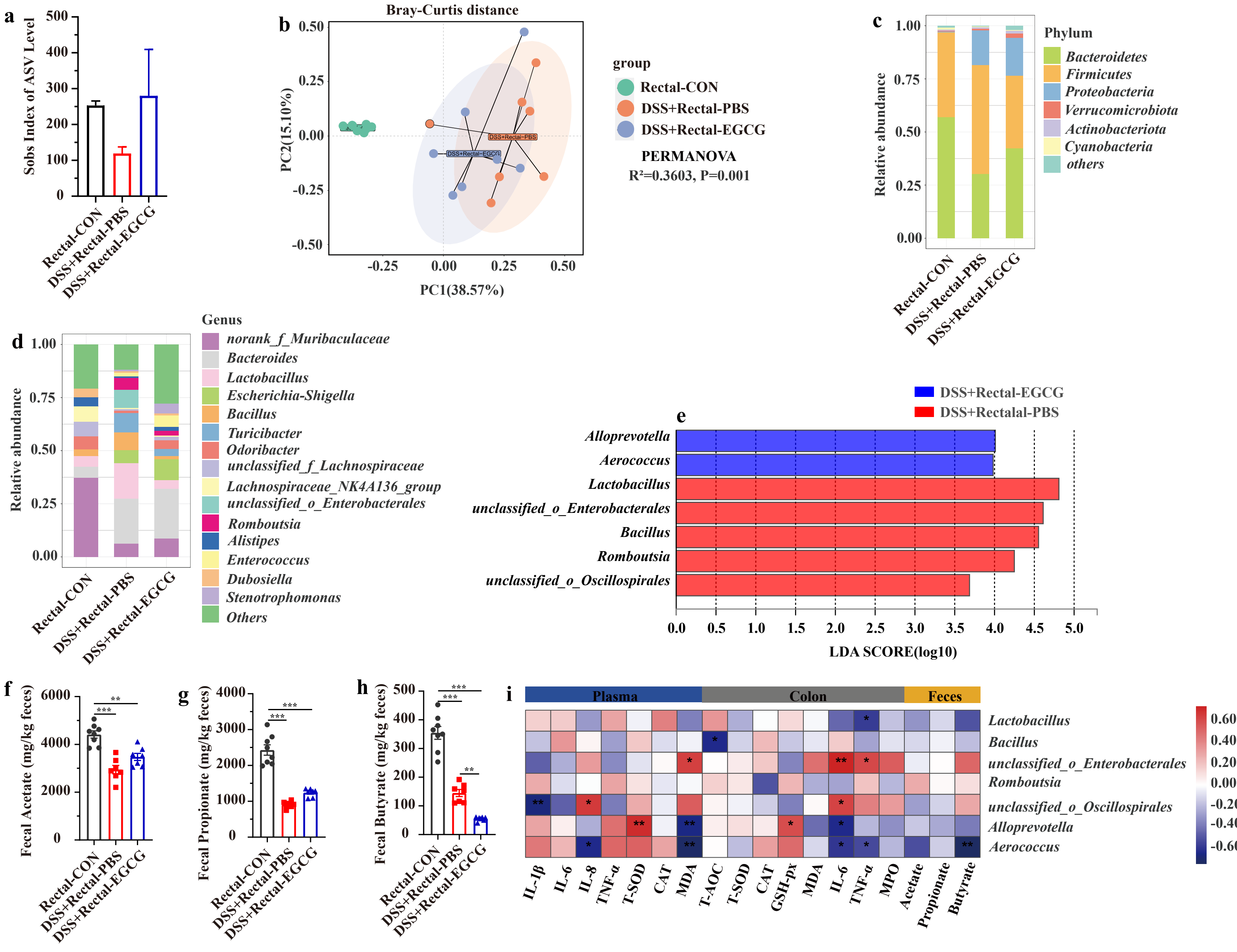

Supplement: Supplementary file 4 — Additional file 3: Figure S3. Oral EGCG regulated the composition and function of intestinal microbiota. (a) α-diversity upon oral therapy represented by the Sobs index. (b) PCoA plots upon rectal therapy assessed by PERMANOVA. The relative abundance of fecal bacterial phyla (c), and genera (d) presented in 99.5% of the community upon rectal therapy. (e) Analysis of differences in the microbial taxa shown by LEfSe (LDA coupled with effect size measurements) upon rectal therapy. Concentrations of fecal acetate (f), propionate (g), and butyrate (h) upon rectal therapy. Data were presented as Means ± SEM (n = 7 per group). Statistical significance was determined using one-way ANOVA, followed by Turkey test. * P ≤ 0.05, ** P ≤ 0.01, *** P ≤ 0.001. (i) Spearman Correlation between intestinal microbiota and anti-inflammatory or anti-oxidative parameters in DSS-treated mice in response to rectal EGCG. The red color denotes a positive correlation, while blue color denotes a negative correlation. The intensity of the color is proportional to the strength of Spearman correlation. * P ≤ 0.05, *** P ≤ 0.001. [file 40168_2021_1115_MOESM3_ESM.pdf]
