# Supplementary material for: Gut microbiota from green tea polyphenol-dosed mice improves intestinal epithelial homeostasis and ameliorates experimental colitis
Source: Microbiome. 2021 Sep 7;9:184. doi: 10.1186/s40168-021-01115-9 (PMC8424887; doi:10.1186/s40168-021-01115-9)

This document contains all the scripts used for the analyses in the microbiota data analysis section.

**Results:**

How was the gut microbiota in colitis mice impacted by EGCG or gut microbiota from EGCG-dosed mice?

The PCoA plots were generated and PERMANOVA was performed based on Bray-Curtis distances and permutations with the Adonis function available.

For generating the PCoA plots, firstly, a function called geom_enterotype used for calculating the confidence interval was generated.

| > geom_enterotype <- function(mapping = NULL, data = NULL, stat = "identity", position = "identity",  alpha = 0.15, prop = 0.6, ..., lineend = "butt", linejoin = "round",  linemitre = 1, arrow = NULL, na.rm = FALSE, parse = FALSE,  nudge_x = 0, nudge_y = 0, label.padding = unit(0.15, "lines"),  label.r = unit(0.15, "lines"), label.size = 0.1,  show.legend = TRUE, inherit.aes = TRUE) {  library(ggplot2)  StatEllipse <- ggproto("StatEllipse", Stat,  required_aes = c("x", "y"),  compute_group = function(., data, scales, level = 0.80, segments = 51, ...) {  library(MASS)  dfn <- 2  dfd <- length(data$x) - 1  if (dfd < 3) {  ellipse <- rbind(c(NA, NA))  } else {  v <- cov.trob(cbind(data$x, data$y))  shape <- v$cov  center <- v$center  radius <- sqrt(dfn * qf(level, dfn, dfd))  angles <- (0:segments) * 2 * pi/segments  unit.circle <- cbind(cos(angles), sin(angles))  ellipse <- t(center + radius * t(unit.circle %*% chol(shape)))  }  ellipse <- as.data.frame(ellipse)  colnames(ellipse) <- c("x", "y")  return(ellipse)  })  GeomEllipse <- ggproto("GeomEllipse", Geom,  draw_group = function(data, panel_scales, coord) {  n <- nrow(data)  if (n == 1)  return(zeroGrob())  munched <- coord_munch(coord, data, panel_scales)  munched <- munched[order(munched$group), ]  first_idx <- !duplicated(munched$group)  first_rows <- munched[first_idx, ]  grid::pathGrob(munched$x, munched$y, default.units = "native",  id = munched$group,  gp = grid::gpar(col = first_rows$colour,  fill = alpha(first_rows$fill, first_rows$alpha), lwd = first_rows$size * .pt, lty = first_rows$linetype))  },  default_aes = aes(colour = NA, fill = "grey20", size = 0.5, linetype = 3, alpha = 0.3, prop = 0.5),  handle_na = function(data, params) {  data  },  required_aes = c("x", "y"),  draw_key = draw_key_path  )    StatConline <- ggproto("StatConline", Stat,  compute_group = function(data, scales) {  library(miscTools)  library(MASS)  df <- data.frame(data$x,data$y)  mat <- as.matrix(df)  center <- cov.trob(df)$center  names(center)<- NULL  mat_insert <- insertRow(mat, 2, center )  for(i in 1:nrow(mat)) {  mat_insert <- insertRow( mat_insert, 2*i, center )  next  }  mat_insert <- mat_insert[-c(2:3),]  rownames(mat_insert) <- NULL  mat_insert <- as.data.frame(mat_insert,center)  colnames(mat_insert) =c("x","y")  return(mat_insert)  },  required_aes = c("x", "y")    )  StatLabel <- ggproto("StatLabel" ,Stat,  compute_group = function(data, scales) {  library(MASS)  df <- data.frame(data$x,data$y)  center <- cov.trob(df)$center  names(center)<- NULL  center <- t(as.data.frame(center))  center <- as.data.frame(cbind(center))  colnames(center) <- c("x","y")  rownames(center) <- NULL  return(center)  },  required_aes = c("x", "y")  )  layer1 <- layer(data = data, mapping = mapping, stat = stat, geom = GeomPoint,  position = position, show.legend = show.legend, inherit.aes = inherit.aes,  params = list(na.rm = na.rm, ...))  layer2 <- layer(stat = StatEllipse, data = data, mapping = mapping, geom = GeomEllipse, position = position, show.legend = FALSE,  inherit.aes = inherit.aes, params = list(na.rm = na.rm, prop = prop, alpha = alpha, ...))  layer3 <- layer(data = data, mapping = mapping, stat = StatConline, geom = GeomPath,  position = position, show.legend = show.legend, inherit.aes = inherit.aes,  params = list(lineend = lineend, linejoin = linejoin,  linemitre = linemitre, arrow = arrow, na.rm = na.rm, ...))  if (!missing(nudge_x) \|\| !missing(nudge_y)) {  if (!missing(position)) {  stop("Specify either `position` or `nudge_x`/`nudge_y`",  call. = FALSE)  }  position <- position_nudge(nudge_x, nudge_y)  }  layer4 <- layer(data = data, mapping = mapping, stat = StatLabel, geom = GeomLabel,  position = position, show.legend = FALSE, inherit.aes = inherit.aes,  params = list(parse = parse, label.padding = label.padding,  label.r = label.r, label.size = label.size, na.rm = na.rm, ...))  return(list(layer1,layer2,layer3,layer4))  } |
| --- |

PCoA plots upon oral therapy. The following workspace contains:

- Oral EGCG matrix.csv: The matrix of samples.
- Oral EGCG group.txt: Samples ID and its corresponding group.

| > setwd("Oral EGCG.file")  > bcdis2 <- read.delim('Oral EGCG matrix.csv', row.names = 1, sep = ',', stringsAsFactors = FALSE, check.names = FALSE)  > group2 <- read.delim('Oral EGCG group.txt', sep = '\t', stringsAsFactors = FALSE)  > library(vegan)  > pcoa2 <- cmdscale(as.dist(bcdis2), k = (nrow(bcdis2) - 1), eig = TRUE)  > summary(pcoa2)  Length Class Mode  points 368 -none- numeric  eig 23 -none- numeric  x 0 -none- NULL  ac 1 -none- numeric  GOF 2 -none- numeric  > pcoa2$eig  [1] 2.310626e+00 8.778665e-01 6.491686e-01 4.322875e-01 3.748503e-01 2.216110e-01 1.841989e-01 1.535450e-01 1.191430e-01  [10] 7.440983e-02 6.717415e-02 5.672498e-02 4.052423e-02 2.208906e-02 1.426320e-02 1.226976e-02 -5.551115e-17 -6.695247e-03  [19] -1.167288e-02 -2.688418e-02 -5.717162e-02 -7.041596e-02 -1.003138e-01  > point2 <- data.frame(pcoa2$point)  > write.csv(point2, 'pcoa.bcdis2_sample.csv')  > pcoa2_eig <- (pcoa2$eig)[1:2] / sum(pcoa2$eig)  > sample_site2 <- data.frame({pcoa2$point})[1:2]  > sample_site2$ID <- rownames(sample_site2)  > names(sample_site2)[1:2] <- c('Baxis1', 'Baxis2')  > sample_site2 <- merge(sample_site2, group2, by = 'ID', all.x = T)  > write.csv(sample_site2, 'bcdis_sample_site2.csv', quote = F)  > oralsite<-read.delim('bcdis_sample_site2.csv',row.names = 1, sep = ',', stringsAsFactors = FALSE, check.names = FALSE)  > oralsite$group <- factor(oralsite$group, levels = c('Oral-CON', 'DSS+Oral-PBS', 'DSS+Oral-EGCG'))  > library(plyr)  > group_border <- ddply(oralsite, 'group', function(df) df[chull(df[[2]], df[[3]]), ])  > library(ggplot2)  > library(RColorBrewer)  > oralpcoa<-ggplot(oralsite, aes(Baxis1, Baxis2, fill=group, label = group))+  geom_enterotype()+  theme(panel.background=element_rect(color = 'black',fill = 'transparent'),panel.grid=element_blank(),  axis.line=element_line(size=0.5,colour='black'))+  geom_vline(xintercept = 0, color = 'gray', size = 0.4,lty=2)+  geom_hline(yintercept = 0, color = 'gray', size = 0.4,lty=2)+  geom_point(shape = 19,size=7,aes(color = group))+  scale_fill_brewer(palette='Set2')+  scale_colour_brewer(palette='Set2')+  labs(x = paste('PC1(', round(100 * pcoa2_eig[1], 2), '%)'),  y = paste('PC2(', round(100 * pcoa2_eig[2], 2), '%)'),title='Bray-Curtis distances')+  theme(plot.title = element_text(size=14,face='bold',hjust = 0.5),  legend.text=element_text(color='black',size=14,face='bold'),  legend.title=element_text(color='black',size=14,face='bold'),  axis.title.y=element_text(color='black',size = 14,face = 'bold'),  axis.title.x=element_text( color='black',size = 14,face = 'bold'),  axis.text.x=element_text( color='black',size = 11,face = 'bold'),  axis.text.y=element_text( color='black',size = 11,face = 'bold'))  > oralpcoa  > ggsave(oralpcoa,filename="oralpcoa.pdf", width =10, height = 7) |
| --- |


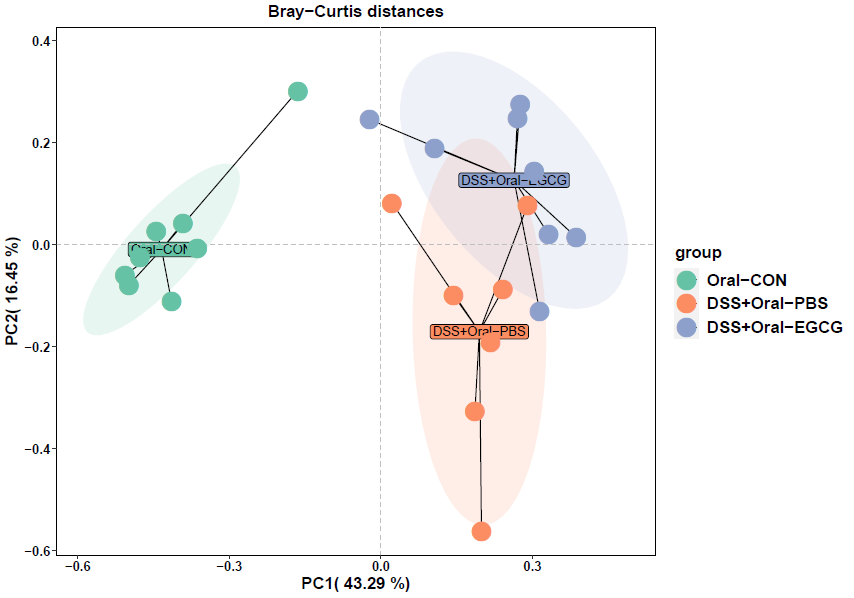


PCoA plots upon rectal therapy. The following workspace contains:

- Rectal EGCG matrix.csv: The matrix of samples.
- Rectal EGCG group.txt: Samples ID and its corresponding group.

| > setwd("Rectal EGCG.file")  > bcdis3 <- read.delim('Rectal EGCG matrix.csv', row.names = 1, sep = ',', stringsAsFactors = FALSE, check.names = FALSE)  > group3 <- read.delim('Rectal EGCG group.txt ', sep = '\t', stringsAsFactors = FALSE)  > library(vegan)  > pcoa3 <- cmdscale(as.dist(bcdis3), k = (nrow(bcdis3) - 1), eig = TRUE)  > summary(pcoa3)  Length Class Mode  points 352 -none- numeric  eig 22 -none- numeric  x 0 -none- NULL  ac 1 -none- numeric  GOF 2 -none- numeric  > pcoa3$eig  [1] 1.898446e+00 7.431494e-01 6.018732e-01 4.644399e-01 2.957317e-01 2.409141e-01 1.737681e-01 1.484015e-01 1.095368e-01  [10] 9.616422e-02 8.998536e-02 6.356356e-02 4.638049e-02 3.812677e-02 1.955112e-02 3.982862e-03 -4.857226e-17 -1.033370e-03  [19] -2.629587e-03 -1.970901e-02 -2.924449e-02 -5.925340e-02  > point3 <- data.frame(pcoa3$point)  > write.csv(point3, 'pcoa.bcdis3_sample.csv')  > pcoa3_eig <- (pcoa3$eig)[1:2] / sum(pcoa3$eig)  > sample_site3 <- data.frame({pcoa3$point})[1:2]  > sample_site3$ID <- rownames(sample_site3)  > names(sample_site3)[1:2] <- c('Baxis1', 'Baxis2')  > sample_site3 <- merge(sample_site3, group3, by = 'ID', all.x = T)  > write.csv(sample_site3, 'bcdis_sample_site3.csv', quote = F)  > rectalsite<-read.delim('bcdis_sample_site3.csv',row.names = 1, sep = ',', stringsAsFactors = FALSE, check.names = FALSE)  > rectalsite$group <- factor(rectalsite$group, levels = c('Rectal-CON', 'DSS+Rectal-PBS', 'DSS+Rectal-EGCG'))  > library(plyr)  > group_border <- ddply(rectalsite, 'group', function(df) df[chull(df[[2]], df[[3]]), ])  > library(ggplot2)  > library(RColorBrewer)  > rectalpcoa<-ggplot(rectalsite, aes(Baxis1, Baxis2, fill=group, label = group))+  geom_enterotype()+  theme(panel.background=element_rect(color = 'black',fill = 'transparent'),panel.grid=element_blank(),  axis.line=element_line(size=0.5,colour='black'))+  geom_vline(xintercept = 0, color = 'gray', size = 0.4,lty=2)+  geom_hline(yintercept = 0, color = 'gray', size = 0.4,lty=2)+  geom_point(shape = 19,size=7,aes(color = group))+  scale_fill_brewer(palette='Set2')+  scale_colour_brewer(palette='Set2')+  labs(x = paste('PC1(', round(100 * pcoa3_eig[1], 2), '%)'),  y = paste('PC2(', round(100 * pcoa3_eig[2], 2), '%)'),title='Bray-Curtis distances')+  theme(plot.title = element_text(size=14,face='bold',hjust = 0.5),  legend.text=element_text(color='black',size=14,face='bold'),  legend.title=element_text(color='black',size=14,face='bold'),  axis.title.y=element_text(color='black',size = 14,face = 'bold'),  axis.title.x=element_text( color='black',size = 14,face = 'bold'),  axis.text.x=element_text( color='black',size = 11,face = 'bold'),  axis.text.y=element_text( color='black',size = 11,face = 'bold'))  > rectalpcoa  > ggsave(rectalpcoa,filename="rectalpcoa.pdf", width =10, height = 7) |
| --- |


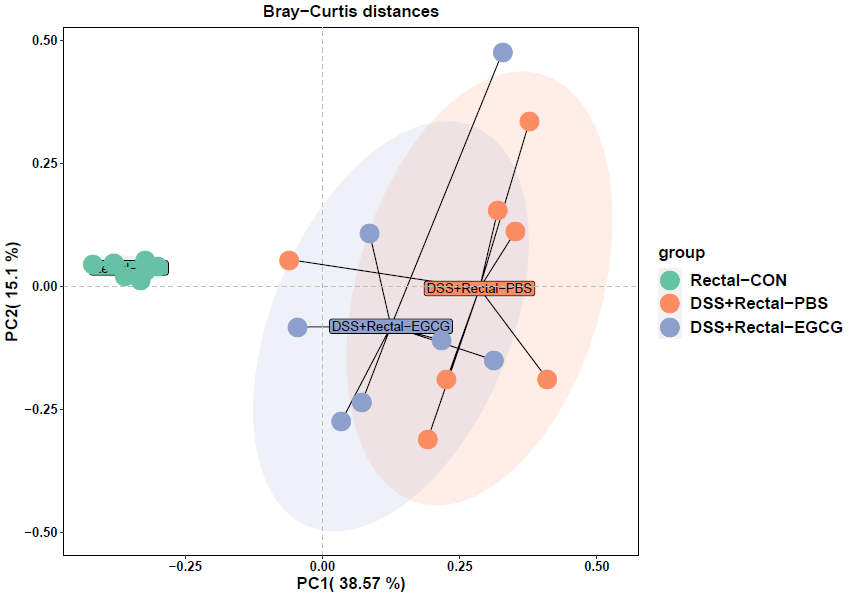


PCoA plots on day 28 among the groups with or without prophylactic EGCG in healthy or colitis mice. The following workspace contains:

- Prophylactic EGCG matrix.csv: The matrix of samples.
- Prophylactic EGCG group.txt: Samples ID and its corresponding group.

| > setwd("Prophylactic EGCG.file")  > bcdis1 <- read.delim(' Prophylactic EGCG matrix.csv', row.names = 1, sep = ',', stringsAsFactors = FALSE, check.names = FALSE)  > group1 <- read.delim('Prophylactic EGCG group.txt', sep = '\t', stringsAsFactors = FALSE)  > library(vegan)  > pcoa1 <- cmdscale(as.dist(bcdis1), k = (nrow(bcdis1) - 1), eig = TRUE)  > summary(pcoa1)  Length Class Mode  points 672 -none- numeric  eig 32 -none- numeric  x 0 -none- NULL  ac 1 -none- numeric  GOF 2 -none- numeric  > pcoa1$eig  [1] 2.111180e+00 5.906602e-01 3.300882e-01 2.827239e-01 2.022418e-01 1.736498e-01 1.497167e-01 1.231309e-01 1.118957e-01  [10] 8.380421e-02 6.895818e-02 5.256310e-02 4.853023e-02 3.177147e-02 2.623054e-02 2.182263e-02 2.062420e-02 1.080841e-02  [19] 5.166198e-03 3.437856e-03 1.878507e-03 -5.551115e-17 -4.901620e-03 -8.429880e-03 -1.597481e-02 -1.835847e-02 -2.800134e-02  [28] -2.953473e-02 -3.388386e-02 -4.448113e-02 -6.013510e-02 -1.207364e-01  > point1 <- data.frame(pcoa1$point)  > write.csv(point1, 'pcoa.bcdis1_sample.csv')  > pcoa1_eig <- (pcoa1$eig)[1:2] / sum(pcoa1$eig)  > sample_site1 <- data.frame({pcoa1$point})[1:2]  > sample_site1$ID <- rownames(sample_site1)  > names(sample_site1)[1:2] <- c('Baxis1', 'Baxis2')  > sample_site1 <- merge(sample_site1, group1, by = 'ID', all.x = T)  > write.csv(sample_site1, 'bcdis_sample_site1.csv', quote = F)  > prosite<-read.delim('bcdis_sample_site1.csv',row.names = 1, sep = ',', stringsAsFactors = FALSE, check.names = FALSE)  > prosite$group <- factor(prosite$group, levels = c('CON', 'DSS', 'EGCG', 'EGCG+DSS'))  > library(plyr)  > group_border <- ddply(prosite, 'group', function(df) df[chull(df[[2]], df[[3]]), ])  > library(ggplot2)  > library(RColorBrewer)  > propcoa<-ggplot(prosite, aes(Baxis1, Baxis2, fill=group, label = group))+  geom_enterotype()+  theme(panel.background=element_rect(color = 'black',fill = 'transparent'),panel.grid=element_blank(),  axis.line=element_line(size=0.5,colour='black'))+  geom_vline(xintercept = 0, color = 'gray', size = 0.4,lty=2)+  geom_hline(yintercept = 0, color = 'gray', size = 0.4,lty=2)+  geom_point(shape = 21,size=7,aes(color = group))+  scale_fill_brewer(palette='Set2')+  scale_colour_brewer(palette='Set2')+  labs(x = paste('PC1(', round(100 * pcoa1_eig[1], 2), '%)'),  y = paste('PC2(', round(100 * pcoa1_eig[2], 2), '%)'),title='Bray-Curtis distances')+  theme(plot.title = element_text(size=14,face='bold',hjust = 0.5),  legend.text=element_text(color='black',size=14,face='bold'),  legend.title=element_text(color='black',size=14,face='bold'),  axis.title.y=element_text(color='black',size = 14,face = 'bold'),  axis.title.x=element_text( color='black',size = 14,face = 'bold'),  axis.text.x=element_text( color='black',size = 11,face = 'bold'),  axis.text.y=element_text( color='black',size = 11,face = 'bold'))  > propcoa  > ggsave(propcoa,filename="propcoa.pdf", width =10, height = 7) |
| --- |


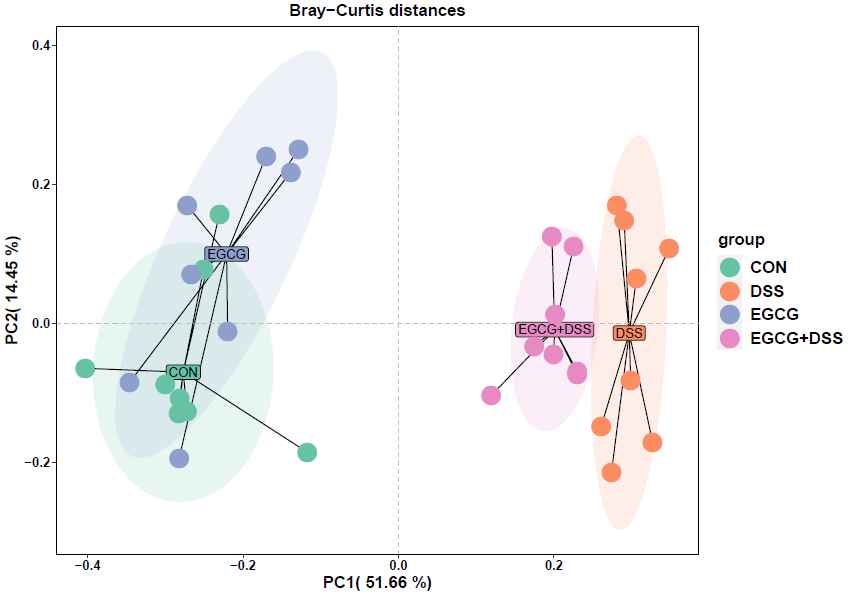


PCoA plots upon FMT or SFF. The following workspace contains:

- EGCG-FMT matrix.csv: The matrix of samples.
- EGCG-FMT group.txt: Samples ID and its corresponding group.

| > setwd("EGCG-FMT.file")  > bcdis4 <- read.delim('EGCG-FMT matrix.csv', row.names = 1, sep = ',', stringsAsFactors = FALSE, check.names = FALSE)  > group4 <- read.delim('EGCG-FMT group.txt', sep = '\t', stringsAsFactors = FALSE)  > library(vegan)  > pcoa4 <- cmdscale(as.dist(bcdis4), k = (nrow(bcdis4) - 1), eig = TRUE)  > summary(pcoa4)  Length Class Mode  points 768 -none- numeric  eig 32 -none- numeric  x 0 -none- NULL  ac 1 -none- numeric  GOF 2 -none- numeric  > pcoa4$eig  [1] 1.999341e+00 1.325607e+00 6.983292e-01 5.692332e-01 4.035428e-01 3.445852e-01 2.740931e-01 2.166828e-01 2.076513e-01  [10] 1.372804e-01 1.265994e-01 8.611983e-02 8.325377e-02 6.486432e-02 5.176271e-02 4.447715e-02 3.177269e-02 2.844783e-02  [19] 1.411030e-02 1.069884e-02 8.466086e-03 1.557811e-03 1.111688e-03 6.938894e-17 -9.722935e-03 -1.405920e-02 -1.783989e-02  [28] -3.035854e-02 -4.149357e-02 -4.437401e-02 -7.574431e-02 -1.104345e-01  > point4 <- data.frame(pcoa4$point)  > write.csv(point4, 'pcoa.bcdis4_sample.csv')  > pcoa4_eig <- (pcoa4$eig)[1:2] / sum(pcoa4$eig)  > sample_site4 <- data.frame({pcoa4$point})[1:2]  > sample_site4$ID <- rownames(sample_site4)  > names(sample_site4)[1:2] <- c('Baxis1', 'Baxis2')  > sample_site4 <- merge(sample_site4, group4, by = 'ID', all.x = T)  > write.csv(sample_site4, 'bcdis_sample_site4.csv', quote = F)  > fmtsite<-read.delim('bcdis_sample_site4.csv',row.names = 1, sep = ',', stringsAsFactors = FALSE, check.names = FALSE)  > fmtsite$group <- factor(fmtsite$group, levels = c('CON-FMT', 'CON-SFF', 'EGCG-FMT','EGCG-SFF'))  > library(plyr)  > group_border <- ddply(fmtsite, 'group', function(df) df[chull(df[[2]], df[[3]]), ])  > library(ggplot2)  > library(RColorBrewer)  > fmtpcoa<-ggplot(fmtsite, aes(Baxis1, Baxis2, fill=group, label = group))+  + geom_enterotype()+  + theme(panel.background=element_rect(color = 'black',fill = 'transparent'),panel.grid=element_blank(),  + axis.line=element_line(size=0.5,colour='black'))+  + geom_vline(xintercept = 0, color = 'gray', size = 0.4,lty=2)+  + geom_hline(yintercept = 0, color = 'gray', size = 0.4,lty=2)+  + geom_point(shape = 19,size=7,aes(color = group))+  + scale_fill_brewer(palette='Set2')+  + scale_colour_brewer(palette='Set2')+  + labs(x = paste('PC1(', round(100 * pcoa4_eig[1], 2), '%)'),  + y = paste('PC2(', round(100 * pcoa4_eig[2], 2), '%)'),title='Bray-Curtis distances')+  + theme(plot.title = element_text(size=14,face='bold',hjust = 0.5),  + legend.text=element_text(color='black',size=14,face='bold'),  + legend.title=element_text(color='black',size=14,face='bold'),  + axis.title.y=element_text(color='black',size = 14,face = 'bold'),  + axis.title.x=element_text( color='black',size = 14,face = 'bold'),  + axis.text.x=element_text( color='black',size = 11,face = 'bold'),  + axis.text.y=element_text( color='black',size = 11,face = 'bold'))  > fmtpcoa  > ggsave(fmtpcoa,filename="fmtpcoa.pdf", width =10, height = 7) |
| --- |


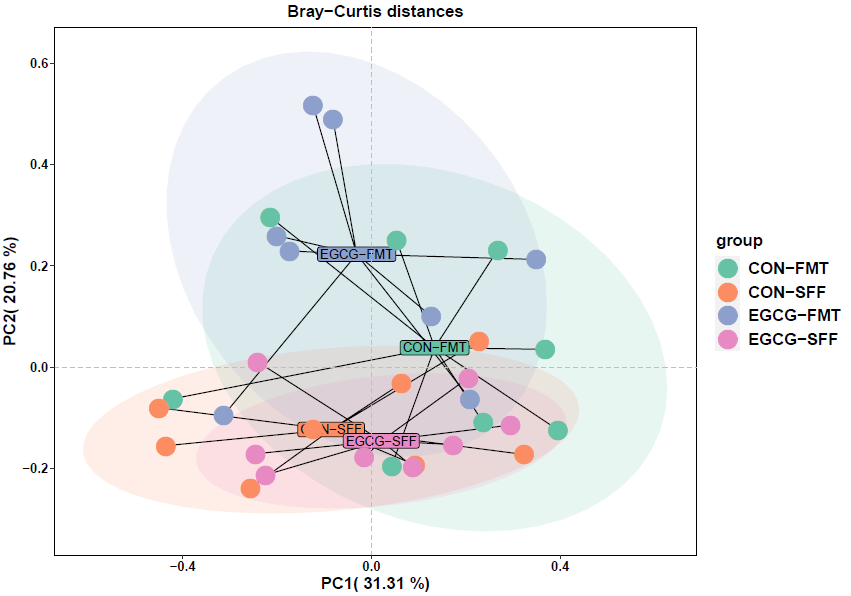


Then, PERMANOVA were performed, with 999 Monte Carlo permutations) based on Bray-Curtis distances and permutations with the Adonis function available. The alternations of community structure among all groups and pairwise were tested.

PERMANOVA analysis upon oral therapy (Figure ). The following workspace contains:

- adoASV2.csv: ASV table created with the percent of sequence dataset (all groups).
- oralgroup.txt: Samples ID and its corresponding group (all groups).
- DEOasv.csv: ASV table created with the percent of sequence dataset (groups with DSS).
- DEOG.txt: Samples ID and its corresponding group (groups with DSS).

For all the three groups:

| > setwd("Oral EGCG")  > allasv <- read.delim('adoASV2.csv', row.names = 1, sep = ',', stringsAsFactors = FALSE, check.names = FALSE)  > allgroup <- read.delim('oralgroup.txt', sep = '\t', stringsAsFactors = FALSE)  > alloralado<-adonis(allasv~allgroup$group,data = allgroup,permutations = 999,method="bray")  > alloralado  Call:  adonis(formula = allasv ~ allgroup$group, data = allgroup, permutations = 999, method = "bray")  Permutation: free  Number of permutations: 999  Terms added sequentially (first to last)  Df SumsOfSqs MeanSqs F.Model R2 Pr(>F)  allgroup$group 2 2.4538 1.22689 5.0915 0.33738 0.001 ***  Residuals 20 4.8194 0.24097 0.66262  Total 22 7.2732 1.00000  ---  Signif. codes: 0 ‘***’ 0.001 ‘**’ 0.01 ‘*’ 0.05 ‘.’ 0.1 ‘ ’ 1 |
| --- |

For the two groups treated with DSS:

| > DEOasv <- read.delim('DEOasv.csv', row.names = 1, sep = ',', stringsAsFactors = FALSE, check.names = FALSE)  > DEOG <- read.delim('DEOG.txt', sep = '\t', stringsAsFactors = FALSE)  > DEOado<-adonis(DEOasv~DEOG$group,data = DEOG,permutations = 999,method="bray")  > DEOado  Call:  adonis(formula = DEOasv ~ DEOG$group, data = DEOG, permutations = 999, method = "bray")  Permutation: free  Number of permutations: 999  Terms added sequentially (first to last)  Df SumsOfSqs MeanSqs F.Model R2 Pr(>F)  DEOG$group 1 0.4669 0.46687 1.8392 0.12394 0.028 *  Residuals 13 3.3000 0.25384 0.87606  Total 14 3.7669 1.00000  ---  Signif. codes: 0 ‘***’ 0.001 ‘**’ 0.01 ‘*’ 0.05 ‘.’ 0.1 ‘ ’ 1 |
| --- |

PERMANOVA analysis upon rectal therapy. The following workspace contains:

- adorectalasv.csv: ASV table created with the percent of sequence dataset (all groups).
- rectalgroup.txt: Samples ID and its corresponding group (all groups).
- DERasv.csv: ASV table created with the percent of sequence dataset (two groups with DSS).
- DERG.txt: ASV table created with the percent of sequence dataset (two groups with DSS).

For all the three groups:

| > setwd("Rectal EGCG")  > allasv <- read.delim('adorectalasv.csv', row.names = 1, sep = ',', stringsAsFactors = FALSE, check.names = FALSE)  > allgroup <- read.delim('rectalgroup.txt', sep = '\t', stringsAsFactors = FALSE)  > allrectalado<-adonis(allasv~allgroup$group,data = allgroup,permutations = 999,method="bray")  > allrectalado  Call:  adonis(formula = allasv ~ allgroup$group, data = allgroup, permutations = 999, method = "bray")  Permutation: free  Number of permutations: 999  Terms added sequentially (first to last)  Df SumsOfSqs MeanSqs F.Model R2 Pr(>F)  allgroup$group 2 2.5079 1.25397 5.3514 0.36033 0.001 ***  Residuals 19 4.4522 0.23433 0.63967  Total 21 6.9602 1.00000  ---  Signif. codes: 0 ‘***’ 0.001 ‘**’ 0.01 ‘*’ 0.05 ‘.’ 0.1 ‘ ’ 1 |
| --- |

For the two groups treated with DSS:

| > DERasv <- read.delim('DERasv.csv', row.names = 1, sep = ',', stringsAsFactors = FALSE, check.names = FALSE)  > DERG <- read.delim('DERG.txt', sep = '\t', stringsAsFactors = FALSE)  > DERado<-adonis(DERasv~DERG$group,data = DERG,permutations = 999,method="bray")  > DERado  Call:  adonis(formula = DERasv ~ DERG$group, data = DERG, permutations = 999, method = "bray")  Permutation: free  Number of permutations: 999  Terms added sequentially (first to last)  Df SumsOfSqs MeanSqs F.Model R2 Pr(>F)  DERG$group 1 0.5387 0.53871 1.8901 0.13608 0.011 *  Residuals 12 3.4202 0.28501 0.86392  Total 13 3.9589 1.00000  ---  Signif. codes: 0 ‘***’ 0.001 ‘**’ 0.01 ‘*’ 0.05 ‘.’ 0.1 ‘ ’ 1 |
| --- |

PERMANOVA analysis on day 28 among the groups with or without prophylactic EGCG in healthy or colitis mice. The following workspace contains:

- adoASV1.csv: ASV table created with the percent of sequence dataset (all groups).
- Exp2group.txt: Samples ID and its corresponding group (all groups).
- CEasv.csv: ASV table created with the percent of sequence dataset (two groups without DSS).
- CEG.txt: Samples ID and its corresponding group (two groups without DSS).
- DEDasv.csv: ASV table created with the percent of sequence dataset (two groups with DSS).
- DEDG.txt: Samples ID and its corresponding group (two groups with DSS).

For all the groups:

| > setwd("prophylacticEGCG")  > allasv <- read.delim('adoASV1.csv', row.names = 1, sep = ',', stringsAsFactors = FALSE, check.names = FALSE)  > allgroup <- read.delim('Exp2group.txt', sep = '\t', stringsAsFactors = FALSE)  > library(vegan)  > allproado<-adonis(allasv~allgroup$group,data = allgroup,permutations = 999,method="bray")  > allproado  Call:  adonis(formula = allasv ~ allgroup$group, data = allgroup, permutations = 999, method = "bray")  Permutation: free  Number of permutations: 999  Terms added sequentially (first to last)  Df SumsOfSqs MeanSqs F.Model R2 Pr(>F)  allgroup$group 3 3.8088 1.26959 8.582 0.47903 0.001 ***  Residuals 28 4.1422 0.14794 0.52097  Total 31 7.9510 1.00000  ---  Signif. codes: 0 ‘***’ 0.001 ‘**’ 0.01 ‘*’ 0.05 ‘.’ 0.1 ‘ ’ 1 |
| --- |

For the two groups:

| > CEasv <- read.delim('CEasv.csv', row.names = 1, sep = ',', stringsAsFactors = FALSE, check.names = FALSE)  > CEG <- read.delim('CEG.txt', sep = '\t', stringsAsFactors = FALSE)  > CEproado<-adonis(CEasv~CEG$group,data = group,permutations = 999,method="bray")  > CEproado  Call:  adonis(formula = CEasv ~ CEG$group, data = group, permutations = 999, method = "bray")  Permutation: free  Number of permutations: 999  Terms added sequentially (first to last)  Df SumsOfSqs MeanSqs F.Model R2 Pr(>F)  CEG$group 1 0.49702 0.49702 2.9299 0.17306 0.001 ***  Residuals 14 2.37492 0.16964 0.82694  Total 15 2.87194 1.00000  ---  Signif. codes: 0 ‘***’ 0.001 ‘**’ 0.01 ‘*’ 0.05 ‘.’ 0.1 ‘ ’ 1 |
| --- |

For the two groups with DSS:

| > DEDasv <- read.delim('DEDasv.csv', row.names = 1, sep = ',', stringsAsFactors = FALSE, check.names = FALSE)  > DEDG <- read.delim('DEDG.txt', sep = '\t', stringsAsFactors = FALSE)  > DEDproado<-adonis(DEDasv~DEDG$group,data = group,permutations = 999,method="bray")  > DEDproado  Call:  adonis(formula = DEDasv ~ DEDG$group, data = group, permutations = 999, method = "bray")  Permutation: free  Number of permutations: 999  Terms added sequentially (first to last)  Df SumsOfSqs MeanSqs F.Model R2 Pr(>F)  DEDG$group 1 0.53207 0.53207 4.2149 0.2314 0.001 ***  Residuals 14 1.76732 0.12624 0.7686  Total 15 2.29939 1.0000  ---  Signif. codes: 0 ‘***’ 0.001 ‘**’ 0.01 ‘*’ 0.05 ‘.’ 0.1 ‘ ’ 1 |
| --- |

PERMANOVA analysis upon FMT. The following workspace contains:

- adofmtasv.csv: ASV table created with the percent of sequence dataset (all groups).
- fmtgroup.txt: Samples ID and its corresponding group (all groups).

For all the groups:

| > setwd("FMT")  > allasv <- read.delim('adofmtasv.csv', row.names = 1, sep = ',', stringsAsFactors = FALSE, check.names = FALSE)  > allgroup <- read.delim('fmtgroup.txt', sep = '\t', stringsAsFactors = FALSE)  > allfmtado<-adonis(allasv~allgroup$group,data = allgroup,permutations = 999,method="bray")  > allfmtado  Call:  adonis(formula = allasv ~ allgroup$group, data = allgroup, permutations = 999, method = "bray")  Permutation: free  Number of permutations: 999  Terms added sequentially (first to last)  Df SumsOfSqs MeanSqs F.Model R2 Pr(>F)  allgroup$group 3 1.5048 0.50161 1.8998 0.16912 0.003 **  Residuals 28 7.3931 0.26404 0.83088  Total 31 8.8979 1.00000  ---  Signif. codes: 0 ‘***’ 0.001 ‘**’ 0.01 ‘*’ 0.05 ‘.’ 0.1 ‘ ’ 1 |
| --- |

For the two groups with FMT:

| > CEFMTasv <- read.delim('CEFMTasv.csv', row.names = 1, sep = ',', stringsAsFactors = FALSE, check.names = FALSE)  > CEFMTG <- read.delim('CEFMTG.txt', sep = '\t', stringsAsFactors = FALSE)  > CEFMTado<-adonis(CEFMTasv~CEFMTG$group,data = CEFMTG,permutations = 999,method="bray")  > CEFMTado  Call:  adonis(formula = CEFMTasv ~ CEFMTG$group, data = CEFMTG, permutations = 999, method = "bray")  Permutation: free  Number of permutations: 999  Terms added sequentially (first to last)  Df SumsOfSqs MeanSqs F.Model R2 Pr(>F)  CEFMTG$group 1 0.4416 0.44163 1.4523 0.09399 0.099 .  Residuals 14 4.2572 0.30409 0.90601  Total 15 4.6989 1.00000  ---  Signif. codes: 0 ‘***’ 0.001 ‘**’ 0.01 ‘*’ 0.05 ‘.’ 0.1 ‘ ’ 1 |
| --- |

For the two groups with SFF:

| > CESFFasv <- read.delim('CESFFasv.csv', row.names = 1, sep = ',', stringsAsFactors = FALSE, check.names = FALSE)  > CESFFG <- read.delim('CESFFG.txt', sep = '\t', stringsAsFactors = FALSE)  > CESFFado<-adonis(CESFFasv~CESFFG$group,data = CESFFG,permutations = 999,method="bray")  > CESFFado  Call:  adonis(formula = CESFFasv ~ CESFFG$group, data = CESFFG, permutations = 999, method = "bray")  Permutation: free  Number of permutations: 999  Terms added sequentially (first to last)  Df SumsOfSqs MeanSqs F.Model R2 Pr(>F)  CESFFG$group 1 0.1945 0.19450 0.97938 0.06538 0.406  Residuals 14 2.7803 0.19859 0.93462  Total 15 2.9748 1.00000 |
| --- |

For the two groups transplanted from normal mice:

| > CFSasv <- read.delim('CFSasv.csv', row.names = 1, sep = ',', stringsAsFactors = FALSE, check.names = FALSE)  > CFSG <- read.delim('CFSG.txt', sep = '\t', stringsAsFactors = FALSE)  > CFSado<-adonis(CFSasv~CFSG$group,data = CFSG,permutations = 999,method="bray")  > CFSado  Call:  adonis(formula = CFSasv ~ CFSG$group, data = CFSG, permutations = 999, method = "bray")  Permutation: free  Number of permutations: 999  Terms added sequentially (first to last)  Df SumsOfSqs MeanSqs F.Model R2 Pr(>F)  CFSG$group 1 0.6446 0.64458 2.433 0.14806 0.015 *  Residuals 14 3.7091 0.26493 0.85194  Total 15 4.3536 1.00000  ---  Signif. codes: 0 ‘***’ 0.001 ‘**’ 0.01 ‘*’ 0.05 ‘.’ 0.1 ‘ ’ 1 |
| --- |

For the two groups transplanted from EGCG-dosed mice:

| > EFSasv <- read.delim('EFSasv.csv', row.names = 1, sep = ',', stringsAsFactors = FALSE, check.names = FALSE)  > EFSG <- read.delim('EFSG.txt', sep = '\t', stringsAsFactors = FALSE)  > EFSado<-adonis(EFSasv~EFSG$group,data = EFSG,permutations = 999,method="bray")  > EFSado  Call:  adonis(formula = EFSasv ~ EFSG$group, data = EFSG, permutations = 999, method = "bray")  Permutation: free  Number of permutations: 999  Terms added sequentially (first to last)  Df SumsOfSqs MeanSqs F.Model R2 Pr(>F)  EFSG$group 1 0.9707 0.97070 4.0829 0.22579 0.001 ***  Residuals 14 3.3285 0.23775 0.77421  Total 15 4.2992 1.00000  ---  Signif. codes: 0 ‘***’ 0.001 ‘**’ 0.01 ‘*’ 0.05 ‘.’ 0.1 ‘ ’ 1 |
| --- |

And, the composition of microbial community was performed.

Firstly, load necessary ecological analysis libraries.

| library(ggplot2)  library(reshape2)  library(stringr)  library("RColorBrewer")  library(cowplot)  library("ggsci") |
| --- |

The relative barplots upon oral EGCG. The following workspace contains:

- oralphylum.txt: the relative abundance on phylum level of single samples.
- oralgenus.txt: the relative abundance on genus level of single samples.

The relative abundance on phylum level upon oral EGCG:

| setwd("Oral EGCG")  phylum2<-read.delim("oralphylum.txt",sep='\t',header=TRUE,stringsAsFactors=F,row.names=1,check.names=F)  phylum2_merged<-aggregate(.~Phylum,phylum2,sum)  write.csv(phylum2_merged, 'phylum2_merged.csv',row.names = F,quote = FALSE)  phylum2_merged<-read.delim("phylum2_merged.csv",sep=',',header=TRUE,stringsAsFactors=FALSE,row.names=1,check.names=T)  phylum2_merged$sum <- rowSums(phylum2_merged)  phylum2_merged <- phylum2_merged[order(phylum2_merged$sum, decreasing = TRUE), ]  phylum2_merged_top5 <- phylum2_merged[1:5, -ncol(phylum2_merged)]  phylum2_merged_top5['Others', ] <- colSums(phylum2_merged) - colSums(phylum2_merged_top5)  write.csv(phylum2_merged_top5, 'phylum2_merged_top5.csv', row.names=T,quote = FALSE)  phylum2_merged_top5<-read.delim("phylum2_merged_top5.csv",sep=',',header=TRUE,stringsAsFactors=FALSE,row.names=1,check.names=T)  phylum2_merged_top5$Taxonomy <- factor(rownames(phylum2_merged_top5), levels = rev(rownames(phylum2_merged_top5)))  phylum2_merged_top5 <-melt(phylum2_merged_top5, id = 'Taxonomy')  group2<-read.delim('oralgroup.txt', sep = '\t', header=TRUE,stringsAsFactors=F)  names(group2)[1] <- 'variable'  phylum2_top5_group <- merge(phylum2_merged_top5, group2, by = 'variable')  phylum2_top5_group$group<-factor(phylum2_top5_group$group,levels=c('Oral-CON','DSS+Oral-PBS','DSS+Oral-EGCG'))  cols1 = brewer.pal(6,"Set3")  oralphylum <- ggplot(phylum2_top5_group, aes(x=group, y=value, fill = Taxonomy)) +  geom_bar(stat="identity",position = 'fill',width = 0.5)+  theme_bw()+scale_fill_manual(values=cols1)+  theme(strip.text=element_text(face="bold",size=12),  panel.spacing = unit(0.4, "lines"),  panel.grid.minor.y = element_line(color="grey", size = 0.4),  panel.background=element_rect(fill="white"))+  labs(x = '', y = 'Relative Abundance')+  guides(fill = guide_legend(reverse = T,ncol = 1,title="Phylum"))+  theme(plot.title = element_text(size=14,face='bold',hjust = 0.5),  legend.position = 'right',  legend.text=element_text(color='black',size=14,face='bold.italic'),  legend.title=element_text(color='black',size=14,face='bold'),  axis.ticks.x=element_blank(),  axis.title.y=element_text(color='black',size = 14,face = 'bold'),  axis.title.x=element_text(color='black',size = 14,face = 'bold'),  axis.text.x=element_text(color='black',size = 14,angle=45,vjust = 1,hjust=1,face = 'bold'),  axis.text.y=element_text(color='black',size = 12,face = 'bold'))  oralphylum  ggsave(oralphylum,filename="oralphylum.pdf", width =6, height =8) |
| --- |


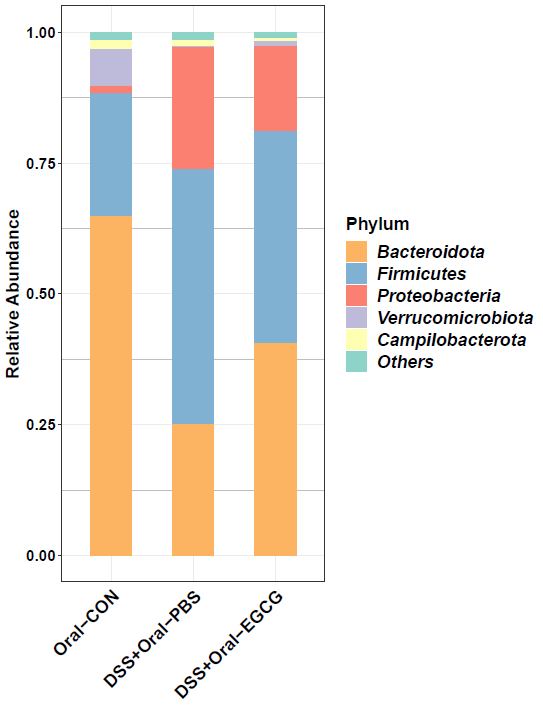


The relative abundance on genus level upon oral EGCG:

| genus2<-read.delim("oralgenus.txt",sep='\t',header=TRUE,stringsAsFactors=F,row.names=1,check.names=F)  genus2_merged<-aggregate(.~Genus,genus2,sum)  write.csv(genus2_merged, 'genus2_merged.csv',row.names = F,quote = FALSE)  genus2_merged<-read.delim("genus2_merged.csv",sep=',',header=TRUE,stringsAsFactors=FALSE,row.names=1,check.names=T)  genus2_merged$sum <- rowSums(genus2_merged)  genus2_merged <- genus2_merged[order(genus2_merged$sum, decreasing = TRUE), ]  genus2_merged_top15 <- genus2_merged[1:15, -ncol(genus2_merged)]  genus2_merged_top15['Others', ] <- colSums(genus2_merged) - colSums(genus2_merged_top15)  write.csv(genus2_merged_top15, 'genus2_merged_top15.csv', row.names=T,quote = FALSE)  genus2_merged_top15<-read.delim("genus2_merged_top15.csv",sep=',',header=TRUE,stringsAsFactors=FALSE,row.names=1,check.names=T)  genus2_merged_top15$Taxonomy <- factor(rownames(genus2_merged_top15), levels = rev(rownames(genus2_merged_top15)))  genus2_merged_top15 <-melt(genus2_merged_top15, id = 'Taxonomy')  group2<-read.delim('oralgroup.txt', sep = '\t', header=TRUE,stringsAsFactors=F)  names(group2)[1] <- 'variable'  genus2_top15_group <- merge(genus2_merged_top15, group2, by = 'variable')  genus2_top15_group$group<-factor(genus2_top15_group$group,levels=c('Oral-CON','DSS+Oral-PBS','DSS+Oral-EGCG'))  cols2 = append(brewer.pal(6,"Accent"),brewer.pal(10,"Set3"))  oralgenus <- ggplot(genus2_top15_group, aes(x=group, y=value, fill = Taxonomy)) +  geom_bar(stat="identity",position = 'fill',width = 0.6)+  theme_bw()+scale_fill_manual(values=cols2)+  theme(strip.text=element_text(face="bold",size=12),  panel.spacing = unit(0.2, "lines"),  panel.grid.minor.y = element_line(color="grey", size = 0.4),  panel.background=element_rect(fill="white"))+  labs(x = '', y = 'Relative Abundance')+  guides(fill = guide_legend(reverse = T,ncol = 1,title="Genus"))+  theme(plot.title = element_text(size=14,face='bold',hjust = 0.5),  legend.position = 'right',  legend.text=element_text(color='black',size=14,face='bold.italic'),  legend.title=element_text(color='black',size=14,face='bold'),  axis.ticks.x=element_blank(),  axis.title.y=element_text(color='black',size = 14,face = 'bold'),  axis.title.x=element_text(color='black',size = 14,face = 'bold'),  axis.text.x=element_text(color='black',size = 14,angle=45,vjust = 1,hjust=1,face = 'bold'),  axis.text.y=element_text(color='black',size = 12,face = 'bold'))  oralgenus  ggsave(oralgenus,filename="oralgenus.pdf", width =8, height =8) |
| --- |


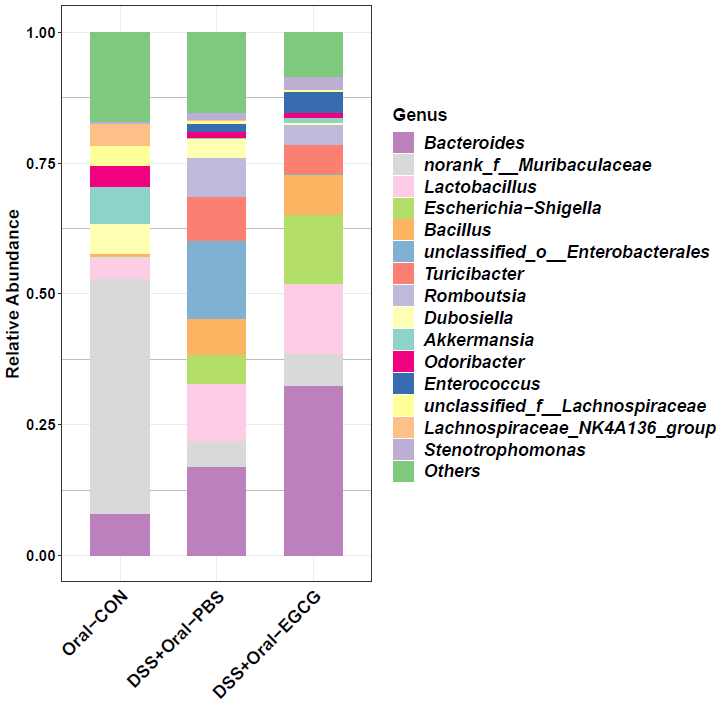


The relative barplots upon rectal EGCG. The following workspace contains:

- Rectalphylum.txt: the relative abundance on phylum level of single samples.
- Rectalgenus.txt: the relative abundance on genus level of single samples.

The relative abundance on phylum level upon rectal EGCG:

| setwd("Rectal EGCG")  phylum3<-read.delim("Rectalphylum.txt",sep='\t',header=TRUE,stringsAsFactors=F,row.names=1,check.names=F)  phylum3_merged<-aggregate(.~Phylum,phylum3,sum)  write.csv(phylum3_merged, 'phylum3_merged.csv',row.names = F,quote = FALSE)  phylum3_merged<-read.delim("phylum3_merged.csv",sep=',',header=TRUE,stringsAsFactors=FALSE,row.names=1,check.names=T)  phylum3_merged$sum <- rowSums(phylum3_merged)  phylum3_merged <- phylum3_merged[order(phylum3_merged$sum, decreasing = TRUE), ]  phylum3_merged_top6 <- phylum3_merged[1:6, -ncol(phylum3_merged)]  phylum3_merged_top6['Others', ] <- colSums(phylum3_merged) - colSums(phylum3_merged_top6)  write.csv(phylum3_merged_top6, 'phylum3_merged_top6.csv', row.names=T,quote = FALSE)  phylum3_merged_top6<-read.delim("phylum3_merged_top6.csv",sep=',',header=TRUE,stringsAsFactors=FALSE,row.names=1,check.names=T)  phylum3_merged_top6$Taxonomy <- factor(rownames(phylum3_merged_top6), levels = rev(rownames(phylum3_merged_top6)))  phylum3_merged_top6 <-melt(phylum3_merged_top6, id = 'Taxonomy')  group3<-read.delim('rectalgroup.txt', sep = '\t', header=TRUE,stringsAsFactors=F)  names(group3)[1] <- 'variable'  phylum3_top6_group <- merge(phylum3_merged_top6, group3, by = 'variable')  phylum3_top6_group$group<-factor(phylum3_top6_group$group,levels=c('Rectal-CON','DSS+Rectal-PBS','DSS+Rectal-EGCG'))  cols1 = brewer.pal(7,"Set3")  rectalphylum <- ggplot(phylum3_top6_group, aes(x=group, y=value, fill = Taxonomy)) +  geom_bar(stat="identity",position = 'fill',width = 0.5)+  theme_bw()+scale_fill_manual(values=cols1)+  theme(strip.text=element_text(face="bold",size=12),  panel.spacing = unit(0.4, "lines"),  panel.grid.minor.y = element_line(color="grey", size = 0.4),  panel.background=element_rect(fill="white"))+  labs(x = '', y = 'Relative Abundance')+  guides(fill = guide_legend(reverse = T,ncol = 1,title="Phylum"))+  theme(plot.title = element_text(size=14,face='bold',hjust = 0.5),  legend.position = 'right',  legend.text=element_text(color='black',size=14,face='bold.italic'),  legend.title=element_text(color='black',size=14,face='bold'),  axis.ticks.x=element_blank(),  axis.title.y=element_text(color='black',size = 14,face = 'bold'),  axis.title.x=element_text(color='black',size = 14,face = 'bold'),  axis.text.x=element_text(color='black',size = 14,angle=45,vjust = 1,hjust=1,face = 'bold'),  axis.text.y=element_text(color='black',size = 12,face = 'bold'))  rectalphylum  ggsave(rectalphylum,filename="rectalphylum.pdf", width =6, height =8) |
| --- |


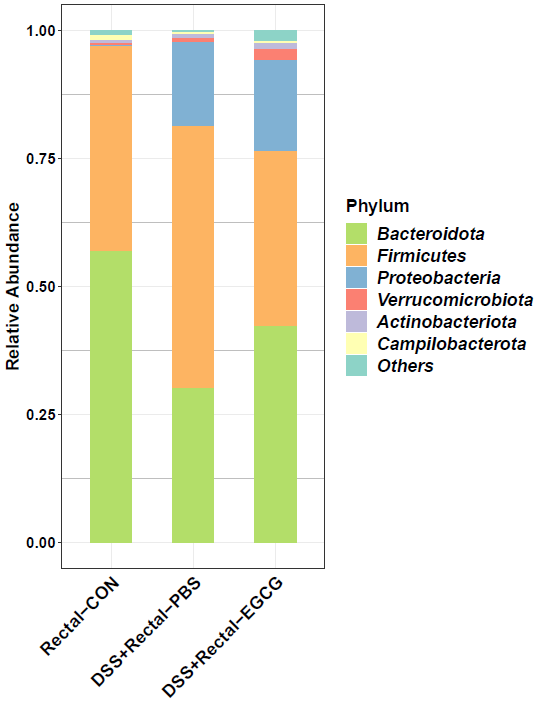


The relative abundance on genus level upon rectal EGCG:

| genus3<-read.delim("rectalgenus.txt",sep='\t',header=TRUE,stringsAsFactors=F,row.names=1,check.names=F)  genus3_merged<-aggregate(.~Genus,genus3,sum)  write.csv(genus3_merged, 'genus3_merged.csv',row.names = F,quote = FALSE)  genus3_merged<-read.delim("genus3_merged.csv",sep=',',header=TRUE,stringsAsFactors=FALSE,row.names=1,check.names=T)  genus3_merged$sum <- rowSums(genus3_merged)  genus3_merged <- genus3_merged[order(genus3_merged$sum, decreasing = TRUE), ]  genus3_merged_top15 <- genus3_merged[1:15, -ncol(genus3_merged)]  genus3_merged_top15['Others', ] <- colSums(genus3_merged) - colSums(genus3_merged_top15)  write.csv(genus3_merged_top15, 'genus3_merged_top15.csv', row.names=T,quote = FALSE)  genus3_merged_top15<-read.delim("genus3_merged_top15.csv",sep=',',header=TRUE,stringsAsFactors=FALSE,row.names=1,check.names=T)  genus3_merged_top15$Taxonomy <- factor(rownames(genus3_merged_top15), levels = rev(rownames(genus3_merged_top15)))  genus3_merged_top15 <-melt(genus3_merged_top15, id = 'Taxonomy')  group3<-read.delim('rectalgroup.txt', sep = '\t', header=TRUE,stringsAsFactors=F)  names(group3)[1] <- 'variable'  genus3_top15_group <- merge(genus3_merged_top15, group3, by = 'variable')  genus3_top15_group$group<-factor(genus3_top15_group$group,levels=c('Rectal-CON','DSS+Rectal-PBS','DSS+Rectal-EGCG'))  cols2 = append(brewer.pal(6,"Accent"),brewer.pal(10,"Set3"))  rectalgenus <- ggplot(genus3_top15_group, aes(x=group, y=value, fill = Taxonomy)) +  geom_bar(stat="identity",position = 'fill',width = 0.6)+  theme_bw()+scale_fill_manual(values=cols2)+  theme(strip.text=element_text(face="bold",size=12),  panel.spacing = unit(0.2, "lines"),  panel.grid.minor.y = element_line(color="grey", size = 0.4),  panel.background=element_rect(fill="white"))+  labs(x = '', y = 'Relative Abundance')+  guides(fill = guide_legend(reverse = T,ncol = 1,title="Genus"))+  theme(plot.title = element_text(size=14,face='bold',hjust = 0.5),  legend.position = 'right',  legend.text=element_text(color='black',size=14,face='bold.italic'),  legend.title=element_text(color='black',size=14,face='bold'),  axis.ticks.x=element_blank(),  axis.title.y=element_text(color='black',size = 14,face = 'bold'),  axis.title.x=element_text(color='black',size = 14,face = 'bold'),  axis.text.x=element_text(color='black',size = 14,angle=45,vjust = 1,hjust=1,face = 'bold'),  axis.text.y=element_text(color='black',size = 12,face = 'bold'))  rectalgenus  ggsave(rectalgenus,filename="rectalgenus.pdf", width =8, height =8) |
| --- |


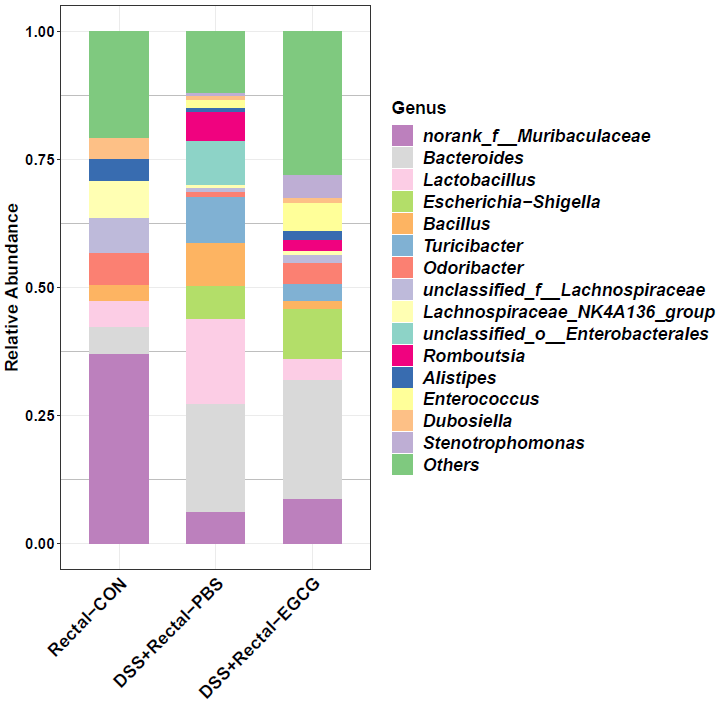


The relative barplots upon prophylactic EGCG. The following workspace contains:

- Exp2phylum.txt: the relative abundance on phylum level of single samples.
- Exp2genus.txt: the relative abundance on genus level of single samples.

The relative abundance on phylum level upon prophylactic EGCG:

| setwd("prophylacticEGCG")  getwd()  phylum1<-read.delim("Exp2phylum.txt",sep='\t',header=TRUE,stringsAsFactors=F,row.names=1,check.names=F)  phylum1_merged<-aggregate(.~Phylum,phylum1,sum)  write.csv(phylum1_merged, 'phylum1_merged.csv',row.names = F,quote = FALSE)  phylum1_merged<-read.delim("phylum1_merged.csv",sep=',',header=TRUE,stringsAsFactors=FALSE,row.names=1,check.names=T)  phylum1_merged$sum <- rowSums(phylum1_merged)  phylum1_merged <- phylum1_merged[order(phylum1_merged$sum, decreasing = TRUE), ]  phylum1_merged_top4 <- phylum1_merged[1:4, -ncol(phylum1_merged)]  phylum1_merged_top4['Others', ] <- colSums(phylum1_merged) - colSums(phylum1_merged_top4)  write.csv(phylum1_merged_top4, 'phylum1_merged_top4.csv', row.names=T,quote = FALSE)  phylum1_merged_top4<-read.delim("phylum1_merged_top4.csv",sep=',',header=TRUE,stringsAsFactors=FALSE,row.names=1,check.names=T)  phylum1_merged_top4$Taxonomy <- factor(rownames(phylum1_merged_top4), levels = rev(rownames(phylum1_merged_top4)))  phylum1_merged_top4 <-melt(phylum1_merged_top4, id = 'Taxonomy')  group1<-read.delim('Exp2group.txt', sep = '\t', header=TRUE,stringsAsFactors=F)  names(group1)[1] <- 'variable'  phylum1_top4_group <- merge(phylum1_merged_top4, group1, by = 'variable')  phylum1_top4_group$group<-factor(phylum1_top4_group$group,levels=c('CON','EGCG','DSS','EGCG+DSS'))  cols1 = brewer.pal(5,"Set3")  prophylum <- ggplot(phylum1_top4_group, aes(x=group, y=value, fill = Taxonomy)) +  geom_bar(stat="identity",position = 'fill',width = 0.5)+  theme_bw()+scale_fill_manual(values=cols1)+  theme(strip.text=element_text(face="bold",size=12),  panel.spacing = unit(0.4, "lines"),  panel.grid.minor.y = element_line(color="grey", size = 0.4),  panel.background=element_rect(fill="white"))+  labs(x = '', y = 'Relative Abundance')+  guides(fill = guide_legend(reverse = T,ncol = 1,title="Phylum"))+  theme(plot.title = element_text(size=14,face='bold',hjust = 0.5),  legend.position = 'right',  legend.text=element_text(color='black',size=14,face='bold.italic'),  legend.title=element_text(color='black',size=14,face='bold'),  axis.ticks.x=element_blank(),  axis.title.y=element_text(color='black',size = 14,face = 'bold'),  axis.title.x=element_text(color='black',size = 14,face = 'bold'),  axis.text.x=element_text(color='black',size = 14,angle=45,vjust = 1,hjust=1,face = 'bold'),  axis.text.y=element_text(color='black',size = 12,face = 'bold'))  prophylum  ggsave(prophylum,filename="prophylum.pdf", width =8, height =8) |
| --- |


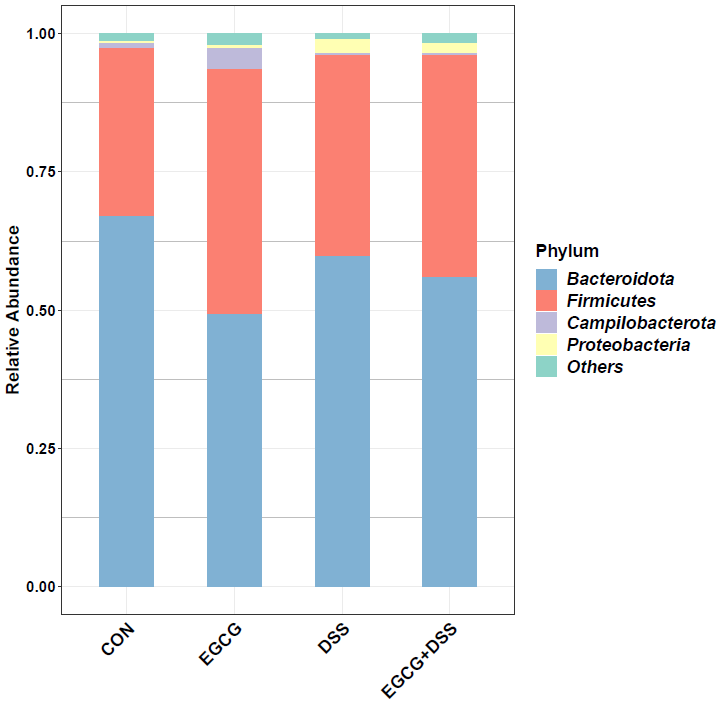


The relative abundance on genus level upon prophylactic EGCG:

| genus1<-read.delim("Exp2genus.txt",sep='\t',header=TRUE,stringsAsFactors=F,row.names=1,check.names=F)  genus1_merged<-aggregate(.~Genus,genus1,sum)  write.csv(genus1_merged, 'genus1_merged.csv',row.names = F,quote = FALSE)  genus1_merged<-read.delim("genus1_merged.csv",sep=',',header=TRUE,stringsAsFactors=FALSE,row.names=1,check.names=T)  genus1_merged$sum <- rowSums(genus1_merged)  genus1_merged <- genus1_merged[order(genus1_merged$sum, decreasing = TRUE), ]  genus1_merged_top15 <- genus1_merged[1:15, -ncol(genus1_merged)]  genus1_merged_top15['Others', ] <- colSums(genus1_merged) - colSums(genus1_merged_top15)  write.csv(genus1_merged_top15, 'genus1_merged_top15.csv', row.names=T,quote = FALSE)  genus1_merged_top15<-read.delim("genus1_merged_top15.csv",sep=',',header=TRUE,stringsAsFactors=FALSE,row.names=1,check.names=T)  genus1_merged_top15$Taxonomy <- factor(rownames(genus1_merged_top15), levels = rev(rownames(genus1_merged_top15)))  genus1_merged_top15 <-melt(genus1_merged_top15, id = 'Taxonomy')  group1<-read.delim('Exp2group.txt', sep = '\t', header=TRUE,stringsAsFactors=F)  names(group1)[1] <- 'variable'  genus1_top15_group <- merge(genus1_merged_top15, group1, by = 'variable')  genus1_top15_group$group<-factor(genus1_top15_group$group,levels=c('CON','EGCG','DSS','EGCG+DSS'))  cols2 = append(brewer.pal(6,"Accent"),brewer.pal(10,"Set3"))  progenus <- ggplot(genus1_top15_group, aes(x=group, y=value, fill = Taxonomy)) +  geom_bar(stat="identity",position = 'fill',width = 0.6)+  theme_bw()+scale_fill_manual(values=cols2)+  theme(strip.text=element_text(face="bold",size=12),  panel.spacing = unit(0.2, "lines"),  panel.grid.minor.y = element_line(color="grey", size = 0.4),  panel.background=element_rect(fill="white"))+  labs(x = '', y = 'Relative Abundance')+  guides(fill = guide_legend(reverse = T,ncol = 1,title="Genus"))+  theme(plot.title = element_text(size=14,face='bold',hjust = 0.5),  legend.position = 'right',  legend.text=element_text(color='black',size=14,face='bold.italic'),  legend.title=element_text(color='black',size=14,face='bold'),  axis.ticks.x=element_blank(),  axis.title.y=element_text(color='black',size = 14,face = 'bold'),  axis.title.x=element_text(color='black',size = 14,face = 'bold'),  axis.text.x=element_text(color='black',size = 14,angle=45,vjust = 1,hjust=1,face = 'bold'),  axis.text.y=element_text(color='black',size = 12,face = 'bold'))  progenus  ggsave(progenus,filename="progenus.pdf", width =8, height =8) |
| --- |


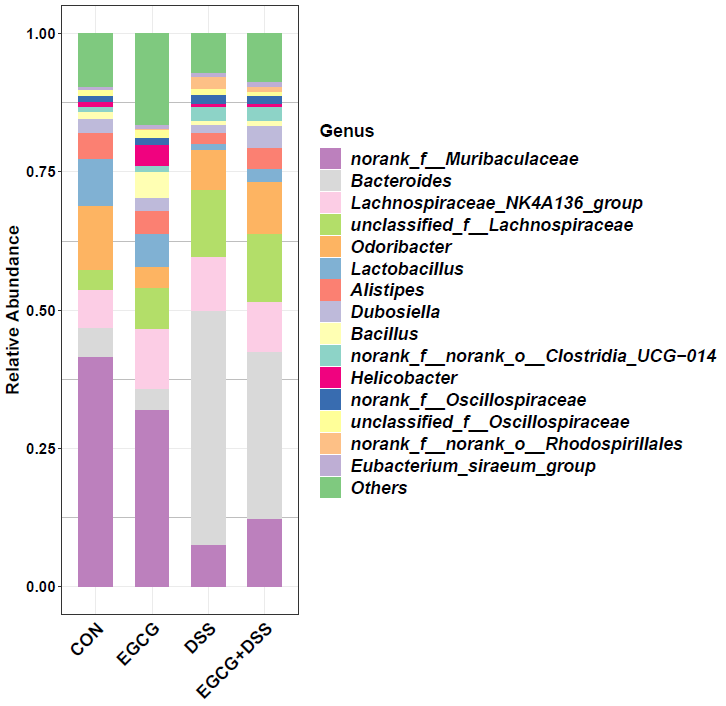


The relative barplots upon FMT or SFF. The following workspace contains:

- FMTphylum.txt: the relative abundance on phylum level of single samples.
- FMTgenus.txt: the relative abundance on genus level of single samples.

The relative abundance on phylum level upon FMT or SFF:

| setwd("FMT")  phylum4<-read.delim("FMTphylum.txt",sep='\t',header=TRUE,stringsAsFactors=F,row.names=1,check.names=F)  phylum4_merged<-aggregate(.~Phylum,phylum4,sum)  write.csv(phylum4_merged, 'phylum4_merged.csv',row.names = F,quote = FALSE)  phylum4_merged<-read.delim("phylum4_merged.csv",sep=',',header=TRUE,stringsAsFactors=FALSE,row.names=1,check.names=T)  phylum4_merged$sum <- rowSums(phylum4_merged)  phylum4_merged <- phylum4_merged[order(phylum4_merged$sum, decreasing = TRUE), ]  phylum4_merged_top6 <- phylum4_merged[1:6, -ncol(phylum4_merged)]  phylum4_merged_top6['Others', ] <- colSums(phylum4_merged) - colSums(phylum4_merged_top6)  write.csv(phylum4_merged_top6, 'phylum4_merged_top6.csv', row.names=T,quote = FALSE)  phylum4_merged_top6<-read.delim("phylum4_merged_top6.csv",sep=',',header=TRUE,stringsAsFactors=FALSE,row.names=1,check.names=T)  phylum4_merged_top6$Taxonomy <- factor(rownames(phylum4_merged_top6), levels = rev(rownames(phylum4_merged_top6)))  phylum4_merged_top6 <-melt(phylum4_merged_top6, id = 'Taxonomy')  group4<-read.delim('FMTgroup.txt', sep = '\t', header=TRUE,stringsAsFactors=F)  names(group4)[1] <- 'variable'  phylum4_top6_group <- merge(phylum4_merged_top6, group4, by = 'variable')  phylum4_top6_group$group<-factor(phylum4_top6_group$group,levels=c('CON-FMT','EGCG-FMT','CON-SFF','EGCG-SFF'))  cols1 = brewer.pal(7,"Set3")  FMTphylum <- ggplot(phylum4_top6_group, aes(x=group, y=value, fill = Taxonomy)) +  geom_bar(stat="identity",position = 'fill',width = 0.5)+  theme_bw()+scale_fill_manual(values=cols1)+  theme(strip.text=element_text(face="bold",size=12),  panel.spacing = unit(0.4, "lines"),  panel.grid.minor.y = element_line(color="grey", size = 0.4),  panel.background=element_rect(fill="white"))+  labs(x = '', y = 'Relative Abundance')+  guides(fill = guide_legend(reverse = T,ncol = 1,title="Phylum"))+  theme(plot.title = element_text(size=14,face='bold',hjust = 0.5),  legend.position = 'right',  legend.text=element_text(color='black',size=14,face='bold.italic'),  legend.title=element_text(color='black',size=14,face='bold'),  axis.ticks.x=element_blank(),  axis.title.y=element_text(color='black',size = 14,face = 'bold'),  axis.title.x=element_text(color='black',size = 14,face = 'bold'),  axis.text.x=element_text(color='black',size = 14,angle=45,vjust = 1,hjust=1,face = 'bold'),  axis.text.y=element_text(color='black',size = 12,face = 'bold'))  FMTphylum  ggsave(FMTphylum,filename="FMTphylum.pdf", width =8, height =8) |
| --- |


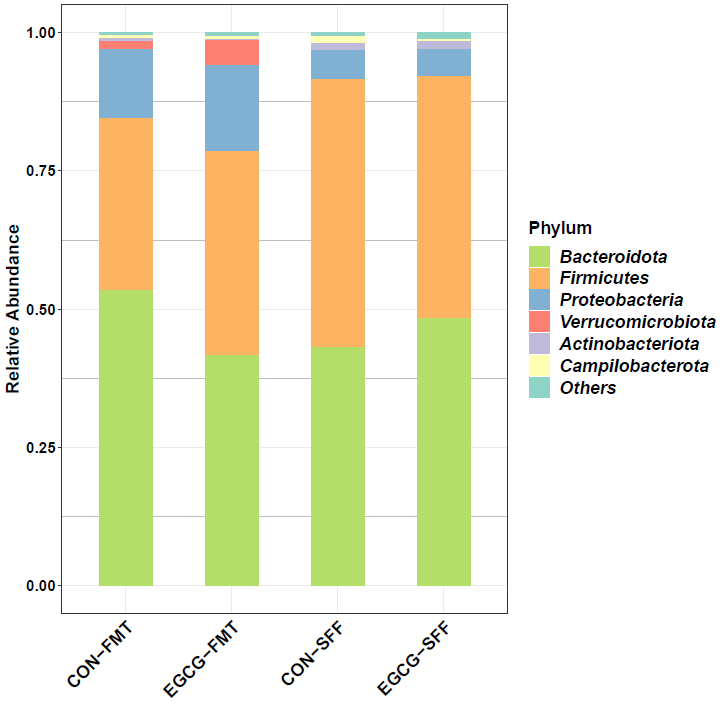


The relative abundance on genus level upon FMT or SFF:

| genus4<-read.delim("FMTgenus.txt",sep='\t',header=TRUE,stringsAsFactors=F,row.names=1,check.names=F)  genus4_merged<-aggregate(.~Genus,genus4,sum)  write.csv(genus4_merged, 'genus4_merged.csv',row.names = F,quote = FALSE)  genus4_merged<-read.delim("genus4_merged.csv",sep=',',header=TRUE,stringsAsFactors=FALSE,row.names=1,check.names=T)  genus4_merged$sum <- rowSums(genus4_merged)  genus4_merged <- genus4_merged[order(genus4_merged$sum, decreasing = TRUE), ]  genus4_merged_top15 <- genus4_merged[1:15, -ncol(genus4_merged)]  genus4_merged_top15['Others', ] <- colSums(genus4_merged) - colSums(genus4_merged_top15)  write.csv(genus4_merged_top15, 'genus4_merged_top15.csv', row.names=T,quote = FALSE)  genus4_merged_top15<-read.delim("genus4_merged_top15.csv",sep=',',header=TRUE,stringsAsFactors=FALSE,row.names=1,check.names=T)  genus4_merged_top15$Taxonomy <- factor(rownames(genus4_merged_top15), levels = rev(rownames(genus4_merged_top15)))  genus4_merged_top15 <-melt(genus4_merged_top15, id = 'Taxonomy')  group4<-read.delim('FMTgroup.txt', sep = '\t', header=TRUE,stringsAsFactors=F)  names(group4)[1] <- 'variable'  genus4_top15_group <- merge(genus4_merged_top15, group4, by = 'variable')  genus4_top15_group$group<-factor(genus4_top15_group$group,levels=c('CON-FMT','EGCG-FMT','CON-SFF','EGCG-SFF'))  cols2 = append(brewer.pal(6,"Accent"),brewer.pal(10,"Set3"))  FMTgenus <- ggplot(genus4_top15_group, aes(x=group, y=value, fill = Taxonomy)) +  geom_bar(stat="identity",position = 'fill',width = 0.6)+  theme_bw()+scale_fill_manual(values=cols2)+  theme(strip.text=element_text(face="bold",size=12),  panel.spacing = unit(0.2, "lines"),  panel.grid.minor.y = element_line(color="grey", size = 0.4),  panel.background=element_rect(fill="white"))+  labs(x = '', y = 'Relative Abundance')+  guides(fill = guide_legend(reverse = T,ncol = 1,title="Genus"))+  theme(plot.title = element_text(size=14,face='bold',hjust = 0.5),  legend.position = 'right',  legend.text=element_text(color='black',size=14,face='bold.italic'),  legend.title=element_text(color='black',size=14,face='bold'),  axis.ticks.x=element_blank(),  axis.title.y=element_text(color='black',size = 14,face = 'bold'),  axis.title.x=element_text(color='black',size = 14,face = 'bold'),  axis.text.x=element_text(color='black',size = 14,angle=45,vjust = 1,hjust=1,face = 'bold'),  axis.text.y=element_text(color='black',size = 12,face = 'bold'))  FMTgenus  ggsave(FMTgenus,filename="FMTgenus.pdf", width =8, height =8) |
| --- |


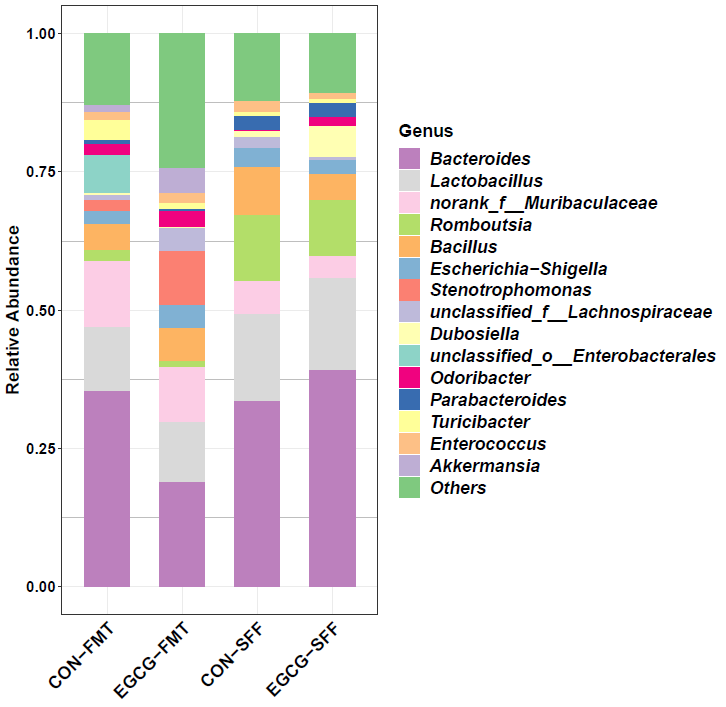


Moreover, the correlation heatmaps between differential bacteria identified by LEfSe analysis and anti-inflammatory or anti-oxidative parameters were performed.

Load necessary ecological analysis packages.

| > library(psych)  > library(pheatmap) |
| --- |

Load necessary file for analysis.

| > setwd("correlation.file") |
| --- |

Correlation analysis upon oral therapy. The following workspace contains:

- oralgenus.csv: the relative abundance of differential bacteria between the two groups with DSS.
- oralenv: anti-inflammatory or anti-oxidative parameters.

| > oralgenus<-read.delim('oralgenus.csv', row.names = 1, sep = ',', stringsAsFactors = FALSE, check.names = FALSE)  > oralenv<-read.delim('oralenv.csv', row.names = 1, sep = ',', stringsAsFactors = FALSE, check.names = FALSE)  > corroral<-corr.test(oralgenus, y = oralenv, use = "pairwise",method="spearman",adjust="none",  alpha=.05,ci=TRUE,minlength=5)  > write.table(corroral$r,"oral.cor.xls",sep="\t",quote=FALSE,col.names=NA)  > write.table(corroral$p,"oral.pvalue.xls",sep="\t",quote=FALSE,col.names=NA)  > library(RColorBrewer)  > col <- colorRampPalette(c("navy","white", "firebrick3"))(100)  > pheatmap(corroral$r, fontsize_number=14,fontsize = 14,cluster_rows = F,  display_numbers = matrix(ifelse(corroral$p <= 0.001 ,"***",  ifelse(corroral$p <= 0.01, "**",  ifelse(corroral$p <= 0.05 ,"*"," "))), nrow(corroral$p)),  cluster_cols = FALSE,fontface = "bold",number_color = "black",border_color = 'grey30',  color = col,angle_col = "45",cellwidth = 24, cellheight = 20) |
| --- |


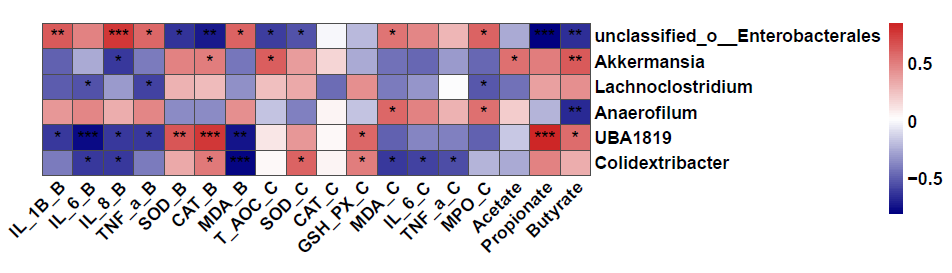


Correlation analysis upon rectal therapy. The following workspace contains:

- rectalgenus.csv: the relative abundance of differential bacteria between the two groups with DSS.
- rectalenv.csv: anti-inflammatory or anti-oxidative parameters.

| > rectalgenus<-read.delim('rectalgenus.csv', row.names = 1, sep = ',', stringsAsFactors = FALSE, check.names = FALSE)  > rectalenv<-read.delim('rectalenv.csv', row.names = 1, sep = ',', stringsAsFactors = FALSE, check.names = FALSE)  > corrrectal<-corr.test(rectalgenus, y = rectalenv, use = "pairwise",method="spearman",adjust="none",  alpha=.05,ci=TRUE,minlength=5)  > write.table(corrrectal$r,"rectal.cor.xls",sep="\t",quote=FALSE,col.names=NA)  > write.table(corrrectal$p,"rectal.pvalue.xls",sep="\t",quote=FALSE,col.names=NA)  > library(RColorBrewer)  > col <- colorRampPalette(c("navy","white", "firebrick3"))(100)  > pheatmap(corrrectal$r, fontsize_number=14,fontsize = 14,cluster_rows = F,  display_numbers = matrix(ifelse(corrrectal$p <= 0.001 ,"***",  ifelse(corrrectal$p <= 0.01, "**",  ifelse(corrrectal$p <= 0.05 ,"*"," "))), nrow(corrrectal$p)),  cluster_cols = FALSE,fontface = "bold",number_color = "black",border_color = 'grey30',  color = col,angle_col = "45", cellwidth = 24, cellheight = 20) |
| --- |


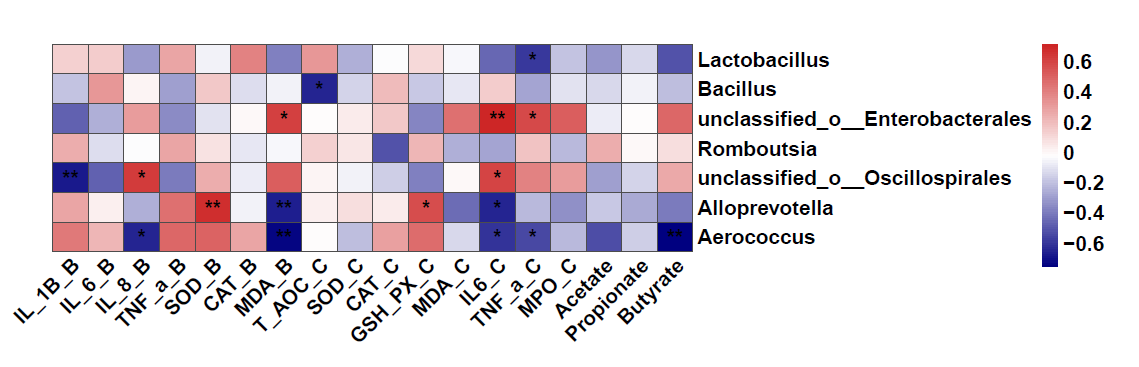


Correlation analysis between healthy groups upon prophylactic EGCG. The following workspace contains:

- pronormalgenus.csv: the relative abundance of differential bacteria between the two healthy groups.
- pronormalenv.csv: anti-inflammatory or anti-oxidative parameters.

| > pronormalgenus<-read.delim('pronormalgenus.csv', row.names = 1, sep = ',', stringsAsFactors = FALSE, check.names = FALSE)  > pronormalenv<-read.delim('pronormalenv.csv', row.names = 1, sep = ',', stringsAsFactors = FALSE, check.names = FALSE)  > corrpronormal<-corr.test(pronormalgenus, y = pronormalenv, use = "pairwise",method="spearman",adjust="none",  + alpha=.05,ci=TRUE,minlength=5)  > write.table(corrpronormal$r,"pronormal.cor.xls",sep="\t",quote=FALSE,col.names=NA)  > write.table(corrpronormal$p,"pronormal.pvalue.xls",sep="\t",quote=FALSE,col.names=NA)  > library(RColorBrewer)  > col <- colorRampPalette(c("navy","white", "firebrick3"))(100)  > pheatmap(corrpronormal$r, fontsize_number=14,fontsize = 14,cluster_rows = F,  display_numbers = matrix(ifelse(corrpronormal$p <= 0.001 ,"***",  ifelse(corrpronormal$p <= 0.01, "**",  ifelse(corrpronormal$p <= 0.05 ,"*"," "))), nrow(corrpronormal$p)),  cluster_cols = FALSE,fontface = "bold",number_color = "black",border_color = 'grey30',  color = col,angle_col = "45",cellwidth = 24, cellheight = 20) |
| --- |


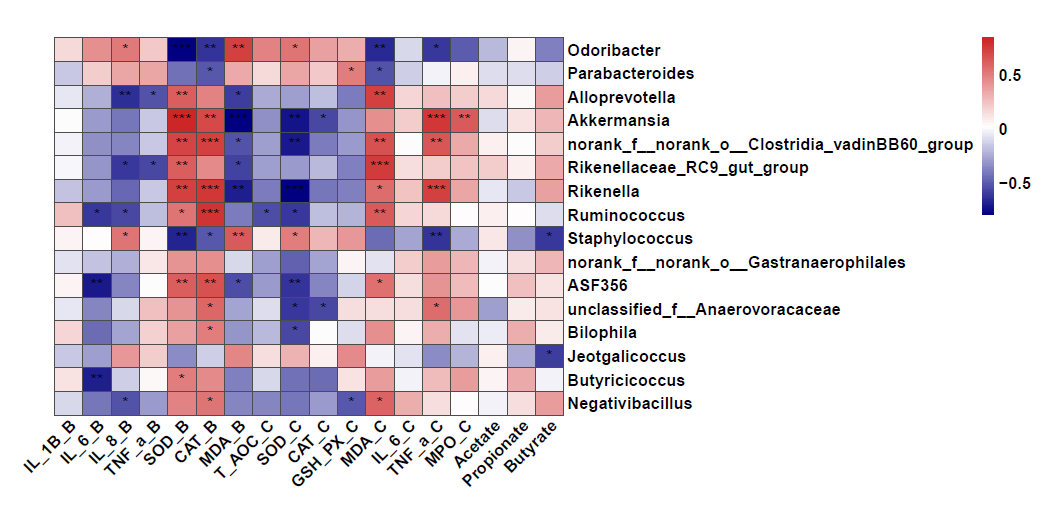


Correlation analysis between colitis groups upon prophylactic EGCG. The following workspace contains:

- prodssgenus.csv: the relative abundance of differential bacteria between the two colitis groups.
- prodssenv.csv: anti-inflammatory or anti-oxidative parameters.

| > prodssgenus<-read.delim('prodssgenus.csv', row.names = 1, sep = ',', stringsAsFactors = FALSE, check.names = FALSE)  > prodssenv<-read.delim('prodssenv.csv', row.names = 1, sep = ',', stringsAsFactors = FALSE, check.names = FALSE)  > corrprodss<-corr.test(prodssgenus, y = prodssenv, use = "pairwise",method="spearman",adjust="none")  > write.table(corrprodss$r,"prodss.cor.xls",sep="\t",quote=FALSE,col.names=NA)  > write.table(corrprodss$p,"prodss.pvalue.xls",sep="\t",quote=FALSE,col.names=NA)  > library(RColorBrewer)  > col <- colorRampPalette(c("navy","white", "firebrick3"))(100)  > pheatmap(corrprodss$r, fontsize_number=14,fontsize = 14,cluster_rows = F,  display_numbers =matrix(ifelse(corrprodss$p <= 0.001 ,"***",  ifelse(corrprodss$p <= 0.01, "**",  ifelse(corrprodss$p <= 0.05 ,"*"," "))), nrow(corrprodss$p)),  cluster_cols = FALSE,fontface= "bold",number_color = "black",border_color = 'grey30',  color = col,angle_col = "45",cellwidth = 24, cellheight = 20) |
| --- |


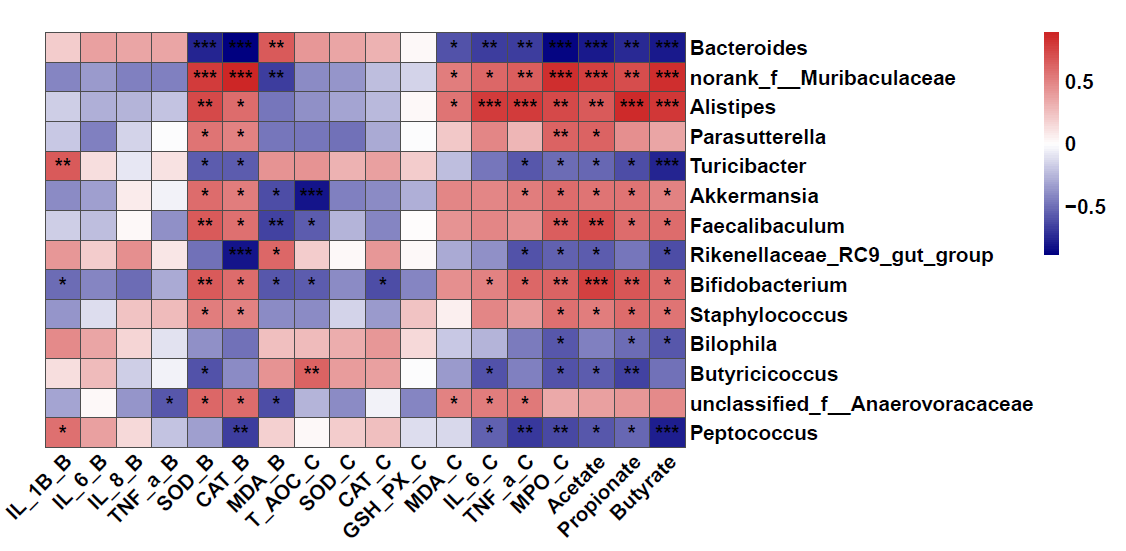


Correlation analysis between groups upon FMT or SFF. The following workspace contains:

- fmtgenus.csv: the relative abundance of differential bacteria between the two colitis groups.
- fmtenv.csv: anti-inflammatory or anti-oxidative parameters.

| > fmtgenus<-read.delim('fmtgenus.csv', row.names = 1, sep = ',', stringsAsFactors = FALSE, check.names = FALSE)  > fmtenv<-read.delim('fmtenv.csv', row.names = 1, sep = ',', stringsAsFactors = FALSE, check.names = FALSE)  > corrfmt<-corr.test(fmtgenus, y = fmtenv, use = "pairwise",method="spearman",adjust="none",  alpha=.05,ci=TRUE,minlength=5)  > write.table(corrfmt$r,"fmt.cor.xls",sep="\t",quote=FALSE,col.names=NA)  > write.table(corrfmt$p,"fmt.pvalue.xls",sep="\t",quote=FALSE,col.names=NA)  > library(RColorBrewer)  > col <- colorRampPalette(c("navy","white", "firebrick3"))(100)  > pheatmap(corrfmt$r, fontsize_number=14,fontsize = 14,cluster_rows = F,  display_numbers = matrix(ifelse(corrfmt$p <= 0.001 ,"***",  ifelse(corrfmt$p <= 0.01, "**",  ifelse(corrfmt$p <= 0.05 ,"*"," "))), nrow(corrfmt$p)),  cluster_cols = FALSE,fontface = "bold",number_color = "black",border_color = 'grey30',  color = col,angle_col = "45", cellwidth = 24, cellheight = 20) |
| --- |


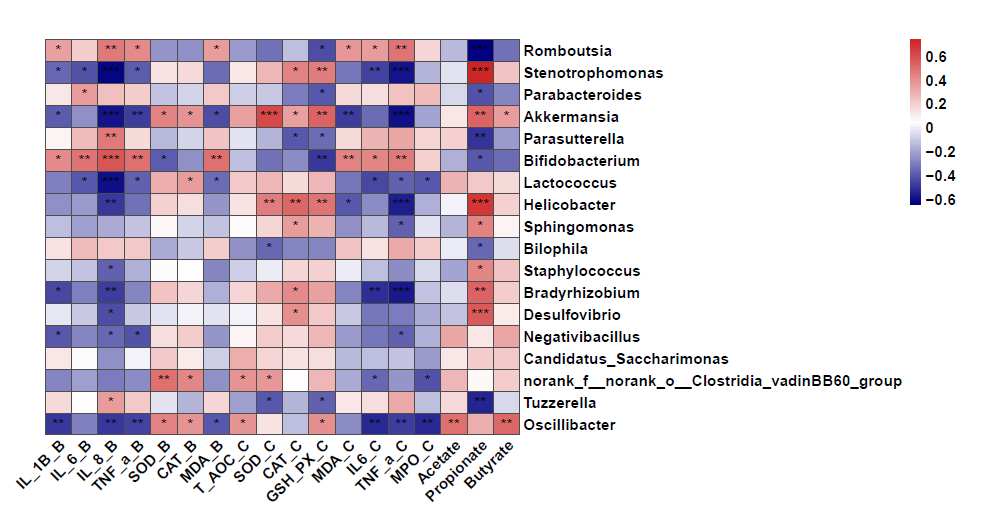

Supplement: Supplementary file 5 — Additional file 4. Full account of statistical analysis performed in R software (version 3.3.1). [file 40168_2021_1115_MOESM4_ESM.docx]
